# Silencing Alpha Synuclein in Mature Nigral Neurons Results in Rapid Neuroinflammation and Subsequent Toxicity

**DOI:** 10.3389/fnmol.2018.00036

**Published:** 2018-02-13

**Authors:** Matthew J. Benskey, Rhyomi C. Sellnow, Ivette M. Sandoval, Caryl E. Sortwell, Jack W. Lipton, Fredric P. Manfredsson

**Affiliations:** ^1^Department of Translational Science and Molecular Medicine, College of Human Medicine, Michigan State University, Grand Rapids, MI, United States; ^2^Mercy Health Saint Mary’s, Grand Rapids, MI, United States

**Keywords:** alpha-synuclein, knockdown, major histocompatibility complex class 1 (MHC-1), microglia, neuroinflammation

## Abstract

Human studies and preclinical models of Parkinson’s disease implicate the involvement of both the innate and adaptive immune systems in disease progression. Further, pro-inflammatory markers are highly enriched near neurons containing pathological forms of alpha synuclein (α-syn), and α-syn overexpression recapitulates neuroinflammatory changes in models of Parkinson’s disease. These data suggest that α-syn may initiate a pathological inflammatory response, however the mechanism by which α-syn initiates neuroinflammation is poorly understood. Silencing endogenous α-syn results in a similar pattern of nigral degeneration observed following α-syn overexpression. Here we aimed to test the hypothesis that loss of α-syn function within nigrostriatal neurons results in neuronal dysfunction, which subsequently stimulates neuroinflammation. Adeno-associated virus (AAV) expressing an short hairpin RNA (shRNA) targeting endogenous α-syn was unilaterally injected into the substantia nigra pars compacta (SNc) of adult rats, after which nigrostriatal pathology and indices of neuroinflammation were examined at 7, 10, 14 and 21 days post-surgery. Removing endogenous α-syn from nigrostriatal neurons resulted in a rapid up-regulation of the major histocompatibility complex class 1 (MHC-1) within transduced nigral neurons. Nigral MHC-1 expression occurred prior to any overt cell death and coincided with the recruitment of reactive microglia and T-cells to affected neurons. Following the induction of neuroinflammation, α-syn knockdown resulted in a 50% loss of nigrostriatal neurons in the SNc and a corresponding loss of nigrostriatal terminals and dopamine (DA) concentrations within the striatum. Expression of a control shRNA did not elicit any pathological changes. Silencing α-syn within glutamatergic neurons of the cerebellum did not elicit inflammation or cell death, suggesting that toxicity initiated by α-syn silencing is specific to DA neurons. These data provide evidence that loss of α-syn function within nigrostriatal neurons initiates a neuronal-mediated neuroinflammatory cascade, involving both the innate and adaptive immune systems, which ultimately results in the death of affected neurons.

## Introduction

A growing body of research suggests that inflammation, mediated by both the innate and adaptive immune systems, is a crucial event in the pathogenesis of Parkinson’s disease (McGeer et al., 1988b; Croisier et al., 2005; Orr et al., 2005; Ouchi et al., 2005; Gerhard et al., 2006; Cebrián et al., 2014b). For example, increased numbers of microglia are present in virtually all brain regions affected by Parkinson’s disease (Ouchi et al., 2005; Gerhard et al., 2006). Microglia within the Parkinson’s disease brain express the major histocompatibility complex (MHC) class II and CD68, indicating that they are activated and phagocytic (McGeer et al., 1988b; Croisier et al., 2005). Parkinson’s disease patients exhibit increased pro-inflammatory cytokines in the brain and periphery (Mogi et al., 1994a,b, 1996), and infiltration of peripheral leukocytes, such as T-cells, into the brain (McGeer et al., 1988a). Within the Parkinson’s disease brain catecholamine neurons express major histocompatibility complex class 1 (MHC-1; McGeer et al., 1988b; Croisier et al., 2005; Orr et al., 2005; Ouchi et al., 2005; Gerhard et al., 2006; Cebrián et al., 2014b), and Lewy bodies and dopamine (DA) neurons label with immunoglobulin G (Orr et al., 2005; Ouchi et al., 2005; Gerhard et al., 2006). Finally, serum or immunoglobulin G isolated from Parkinson’s disease patients can mediate selective destruction of DA neurons in models of Parkinson’s disease (McGeer et al., 1988b; Defazio et al., 1994; Chen et al., 1998; Croisier et al., 2005).

Whether neuroinflammation is an active participant in neurodegeneration or merely a reactionary response to cell death remains contentious. Microglia are increased within disease-affected brain regions of early stage Parkinson’s disease patients (Mogi et al., 1994a,b, 1996; Gerhard et al., 2006), and increased microglia remain constant over the disease duration (McGeer et al., 1988a; Croisier et al., 2005; Gerhard et al., 2006). These data suggest that an initial immunogenic signal triggers microglial activation and recruitment to disease affected brain regions early in Parkinson’s disease, prior to cell death. Aberrations in the expression or function of the protein alpha synuclein (α-syn) may be one such immunogenic signal, capable of directly initiating an inflammatory cascade.

α-syn is a small protein that is expressed throughout many tissues in the body, but is highly enriched within neural tissue, where it primarily localizes to the presynaptic terminal (Maroteaux et al., 1988; Iwai et al., 1995). Missense mutations or multiplications of the α-syn gene cause familial Parkinson’s disease (Polymeropoulos et al., 1997; Singleton et al., 2003), and aggregated α-syn protein is a primary component of Lewy bodies and Lewy neurites (Spillantini et al., 1997), the defining histopathological features of idiopathic Parkinson’s disease. Further, perturbations in α-syn homeostasis can initiate an inflammatory response. α-syn depositions correlate, both spatially and temporally, with the presence of activated microglia (Croisier et al., 2005), and antibodies against α-syn have been isolated from the serum and cerebrospinal fluid of Parkinson’s disease patients (Papachroni et al., 2007). Activated microglia are also found surrounding Lewy bodies in other synucleinopathies, suggesting that neuroinflammation is a common response to α-syn dysfunction (Streit and Xue, 2016). Supporting this idea, neuroinflammation is a common feature reported in virtually all α-syn-based animals models (reviewed in Magen and Chesselet, 2010; Sanchez-Guajardo et al., 2013). Within rodents, transgenic or virally mediated overexpression of α-syn, or injection of recombinant α-syn protein, cause microgliosis, pro-inflammatory cytokine production, and infiltration of peripheral T-cells within the brain (Theodore et al., 2008; Wilms et al., 2009; Sanchez-Guajardo et al., 2010, 2013; Barkholt et al., 2012; Watson et al., 2012; Fischer et al., 2016). Mimicking the human disease, microgliosis reported in α-syn based animal models occurs during neuronal dysfunction, but prior to cell death (Theodore et al., 2008; Sanchez-Guajardo et al., 2010), suggesting that the inflammatory response actively contributes to neurodegeneration.

An important question that remains unanswered is how perturbations in α-syn homeostasis initiate neuroinflammation. Currently, the prevailing hypothesis posits that α-syn is released from neurons (either actively or following cell death) after which it directly stimulates glia, thereby initiating the inflammatory process. Supporting this theory, recombinant α-syn or conditioned media from α-syn expressing cells stimulates a pro-inflammatory response in cultured microglia (Su et al., 2008; Lee et al., 2010; Kim et al., 2016). However, it appears that loss of normal α-syn function is also capable of eliciting a pro-inflammatory response without a direct interaction between α-syn and glia. For example, microglia isolated from α-syn germline knockout (KO) mice display increased reactivity, both basally and following stimulus, as well as increased cytokine secretion (Austin et al., 2006, 2011). Bidirectional crosstalk between neurons and glia is an integral component to both the normal homeostatic maintenance of healthy neurons and the phagocytic removal of damaged neurons (Sheridan and Murphy, 2013). Thus, we hypothesized that loss of *normal* α-syn function, resulting from aggregation or genetic silencing (Benskey et al., 2016a), leads to neuronal dysfunction and subsequent stimulation of microgliosis and inflammation. We aimed to test this hypothesis without the confounding variable of increased extracellular α-syn, which is able to directly stimulate microglia. Endogenous or overexpressed α-syn is secreted from neurons (Lee et al., 2005; Jang et al., 2010), while injection of recombinant α-syn protein directly exposes microglia to α-syn (Yu et al., 2010). Thus, to induce intra-neuronal α-syn dysfunction without directly increasing microglial exposure to α-syn, we chose to silence endogenous α-syn expression within mature nigrostriatal neurons, using adeno-associated virus (AAV) expressing a short hairpin RNA (shRNA) targeting endogenous rat α-syn.

Removal of α-syn from mature nigrostriatal neurons of adult rodents and non-human primates results in neurodegeneration that recapitulates many neuropathological aspects of Parkinson’s disease (Gorbatyuk et al., 2010; Khodr et al., 2011, 2014; Benskey et al., 2016a; Collier et al., 2016). However, the time course of neuronal dysfunction and degeneration following α-syn knockdown has never been thoroughly characterized. Thus, the purpose of this study was to: (1) establish a time line of nigrostriatal neuronal dysfunction and degeneration following removal of endogenous α-syn; and (2) examine the effects of intra-neuronal α-syn dysfunction on neuroinflammation.

## Materials and Methods

### cDNA Clones and Virus Production

α-syn (GAAGGACCAGATGGGCAAG), myocardin (TGCAACTGCAGAAGCAGAA), or scrambled (CACAAGATGAAGAGCACCA) siRNAs were designed using standard algorithms (Toro Cabrera and Mueller, 2016), and sequences were confirmed against available genomic data in order to ensure α-syn specificity. shRNAs were expressed by the H1 promoter (Benskey et al., 2014). All vectors also contained a green fluorescent protein (GFP) reporter gene under control of the hybrid chicken β-actin/cytomegalovirus promoter.

AAV was packaged through the co-transfection of human embryonic kidney 293T (HEK293T) cells with the AAV genome and an AAV5 helper plasmid. AAV was isolated from cellular lysates using an iodixanol step gradient and purified via column chromatography using a Q sepharose column (Amersham Biosciences). Virus titers were determined by dot blot and expressed in viral genomes/milliliter (vg/ml; Benskey et al., 2016b). Throughout the course of this study, several independent lots of the respective AAV vectors were created and used as needed. Results remained consistent between vector lots.

Lentivirus (LV) was packaged through the co-transfection of HEK293T cells with the respective LV genome, a LV packaging vector and a vesicular stomatitis virus glycoprotein psuedotyping plasmid. LV was concentrated from cellular media via ultracentrifugation, and resuspended in Dulbeccos modified eagles medium (Benskey and Manfredsson, 2016b). Viral titers were determined using the Lenti-X-qRT PCR kit (Clonetech).

### Animals and Surgery

Experiments involving animals were conducted in accordance with the Michigan State University Institutional Animal Care and Use Committee (AUF 10/15-156-00). The protocol was approved by the Michigan State University Institutional Animal Care and Use Committee. Rat experiments were conducted using young adult (220 g) male Sprague-Dawley rats. Mouse experiments were conducted using young adult (30 g) male α-syn knockout (B6:129X1-SNCA^tmRosl/^J; Abeliovich et al., 2000) or wild type C57Bl6/j mice. Rats were housed two per cage while mice were housed four per cage. All animals were maintained in a light-controlled (12 h light/dark cycle; lights on at 06:00 h) room, and provided food and water *ad libitum*.

Surgery was performed under 2% isoflurane anesthesia as previously described (Benskey and Manfredsson, 2016a). AAV expressing the α-syn or myocardin shRNAs were injected into the rat substantia nigra pars compacta (SNc) or cerebellum at a titer of 2.6 × 10^12^ vg/ml (Full) or 1.3 × 10^12^ vg/ml (Half) in the same volume. AAV expressing the α-syn or scrambled shRNAs were injected into the mouse SNc at a titer of 3 × 10^13^vg/ml. All stereotaxic coordinates were calculated relative to Bregma. Unilateral 1.5 μl injections to the rat SNc: −5.4 mm anterior/posterior; +2.0 mm medial/lateral; −7.2 mm dorsal ventral (relative to dura). Unilateral 1.5 μl injections to the rat cerebellum: −11.3 mm anterior/posterior; +2.5 mm medial/lateral; −6.2 mm dorsal/ventral (relative to dura). Unilateral 1 μl injections to the mouse SNc: −3 mm anterior/posterior; +1.5 mm medial/lateral; −4.6 mm dorsal ventral (relative to dura). Injections were performed using an automated micropump (World Precision Instruments).

### Cell Culture

Undifferentiated PC12 cells were maintained in RPMI-1640 medium supplemented with 10% horse serum, 2.5% fetal bovine serum and 1% penicillin/streptomycin. All cells were grown in a sterile incubator with 5% CO_2_ at 37°C and subcultured every 2–3 days.

### Tissue Collection and Processing

α-syn shRNA toxicity in the rat nigrostriatal system was analyzed at 7, 10, 14, and 21-days post-surgery. α-syn shRNA toxicity in the rat cerebellum or mouse SNc, was analyzed 1-month post-surgery. Animals were sacrificed by a lethal dose of pentobarbital and transcardially perfused with 0.9% saline. The striatum was dissected and immediately frozen in liquid nitrogen for protein and neurochemical analysis. For histological analysis, brains were post fixed in 4% paraformaldehyde, and coronal sections (40 μm) were cut through the midbrain or cerebellum using a microtome. To control for variations in inter-sample processing, the contralateral (uninjected) hemisphere is used as an internal control, and data is presented as percent of the contralateral hemisphere.

### Histology

Free floating immunohistochemistry or immunofluorescence was performed by washing tissue in TBS containing 0.25% Triton-X 100 (TBS-Tx), blocking in 10% normal goat serum, and incubating in primary antibody over night at room temperature. Primary antibodies used: mouse anti-tyrosine hydroxylase (TH; Millipore MAB318), rabbit anti-TH (Millipore AB152), mouse anti-alpha-synuclein (BD Transduction Laboratories 610787) rabbit anti-calbindin (AbCam ab11426), mouse anti-HUc/d (Invitrogen A-21271), mouse anti-neuronal nuclear protein (NeuN; Millipore MAB377), rabbit anti-ionized calcium-biding adapter molecule 1 (IBA1; Wako 019-19741), rabbit anti-CD68 (Abcam ab31630), mouse anti-rat MHC-1 (Bio Rad MCA51GA), mouse anti-rat CD3 (Bio Rad MCA772), rabbit anti-cleaved caspase-3 (cell signaling 9661), and rabbit anti-vesicular monoamine transporter (VMAT; Abcam ab81855).

For immunohistochemical detection, tissue was washed in TBS-Tx and incubated in a biotin-conjugated goat anti-rabbit (Abcam ab6720), or a goat anti-mouse (Millipore AP124B) secondary antibody for 2 h at room temperature. Bound peroxidase was visualized with 0.05% 3-3′-diaminobenzidine tetrahydrochloride (Sigma) with 0.01% hydrogen peroxide using an ABC Elite kit (Vector Laboratories). For immunfluorescent detection, tissue was washed in TBS-Tx and incubated with alexafluor 488 (Invitrogen), alexafluor 594 (Invitrogen) or LiCor 680 (LiCor-Biosciences) secondary antibodies targeting the respective primary antibody host species.

### Densitometry

Quantification of TH and VMAT protein levels in the striatum was made indirectly using an Odyssey near infrared scanner (Manfredsson et al., 2009; Benskey et al., 2016b). Briefly, striatal tissue was processed for immunodetection of TH and VMAT as described above, after which tissue was incubated in goat anti-mouse LiCor 680LT (925-68020) or goat anti-rabbit 800CW (925-32211) secondary antibodies. Tissue was scanned using the Odyssey infrared scanner using standardized scanning intensities optimized to avoid saturation. Image Studio software 5.2. (Li-COR Biosciences) was used to quantify signal intensity within the ipsilateral and contralateral striata. Signal was normalized to background (cortex), quantified as total intensity per unit area and expressed as the average signal from the ipsilateral hemisphere as a percent of the average signal from contralateral hemisphere.

### Western Blot Analysis

As previously described (Benskey et al., 2013), PC12 cells were collected and homogenized in ice-cold lysis buffer (TBS containing 1% SDS, 1 mM DTT with Complete Mini Protease Inhibitor Cocktail Tablet (Roche)). Supernatants containing total cytosolic protein were assayed for protein content using the BCA assay. Protein (30 μg) was run on a 4%–20% polyacrylamide gel and transferred to a 0.2 μm nitrocellulose membrane. For detection of endogenous α-syn, membranes were then incubated in 0.4% paraformaldehyde for 30 min at room temperature. Membranes were blocked with 5% non-fat dry milk and incubated with mouse anti-alpha synuclein (BD Transduction Laboratories 610787), rabbit anti-beta synuclein (AbCam ab76111) and mouse anti-GAPDH (Cell Signaling D16H11) primary antibodies overnight at 4°C. Membranes were then incubated with goat anti-mouse LiCor 680LT (925-68020) or goat-anti mouse LiCor 800CW (925-32210). Membranes were visualized with the Odyssey infrared imager (Li-Cor Bioscience) and protein band density was quantified using Image Studio software 5.2.

### Neurochemistry

Striatal tissue was homogenized in antioxidant solution and a portion of the homogenate was used for protein determination using the BCA assay (Manfredsson et al., 2007). The remaining homogenate was centrifuged at 14,000× *g* for 20 min and supernatants were used for high performance liquid chromatography to detect DA and 3,4-dihydroxyphenylacetic acid (DOPAC) as previously described (Koprich et al., 2003). Calculated concentrations of catecholamines were normalized to protein content to account for variations in sample size.

### Unbiased Stereology and Manual Cell Counting

Unbiased stereological estimates were performed as previously described (Benskey et al., 2016b). Using Stereo-Investigator software (Version 4.03; Microbrightfield, Inc., Williston, VT, USA, 2000), sections were viewed on a screen at low magnification (4×) and the region of interest was delineated through the rostrocaudal extent of the respective nuclei. Every sixth section was sampled using the optical fractionator method. Counting of cells was performed using a 60× oil objective on an Olympus BX53 microscope equipped with a motorized stage. The coefficient of error for each estimate was calculated and was less than 0.1 (Gundersen, *m* = 1). The number of purkinje cells in lobule 3 of the cerebellar vermis (3Cb) was quantified by manually counting nissl+ cells located within the neuroanatomical limits of the purkinje cell layer (PCL), with a round morphology and somal diameter of least 16 μm. The large size of purkinje soma excludes other local cell types from the analysis. 16 μm was chosen based on an analysis of 50 calbindin+ purkinje cells. From these 50 cells, the average, minimum and maximum cross sectional somal diameter was 19.24 μm, 15.82 μm and 24.67 μm, respectively. Purkinje cells in the 3Cb were quantified within three adjacent sections at the level of the injection site (−11.3 mm anterior/posterior), and the mean number of purkinje cells was averaged over all sections within a single animal.

To quantify the number of MHC-1 positive neurons in the ventral midbrain, midbrain tissue was processed for immunofluorescent detection (as described above) of rat MHC-1. High magnification images of the SNc were obtained from four sections spanning the rostral-caudal axis of the ventral midbrain. The ImageJ cell counter plugin was used to manually count the number of MHC-1 expressing neurons from photomicrographs. To ensure quantification of MHC-1+ neurons, and exclude MHC-1+ microglia from the analysis, a priori criteria were set based on measurements obtained from 50 MHC-1+ cells that co-express the pan neuronal marker HUc/d (see Figures 4Y–CC). From these 50 cells, the average, minimum and maximum cross sectional somal diameter was 20.69 μm, 11.17 μm and 33.15 μm, respectively. Thus, inclusionary criteria stated that cells must: be MHC-1+, have a distinctive cell boundary, have a clearly discernable nucleus, have a somal diameter of at least 11 μm at its widest cross-sectional distance (this somal diameter excludes microglia which had an average cross-sectional somal diameter of 7.8 μm), and have no more than two processes to be included in the analysis. The mean number of MHC-1+ neurons was averaged over all sections within a single animal and presented as MHC-1+ neurons per ipsilateral SNc section.

**Figure 1:**
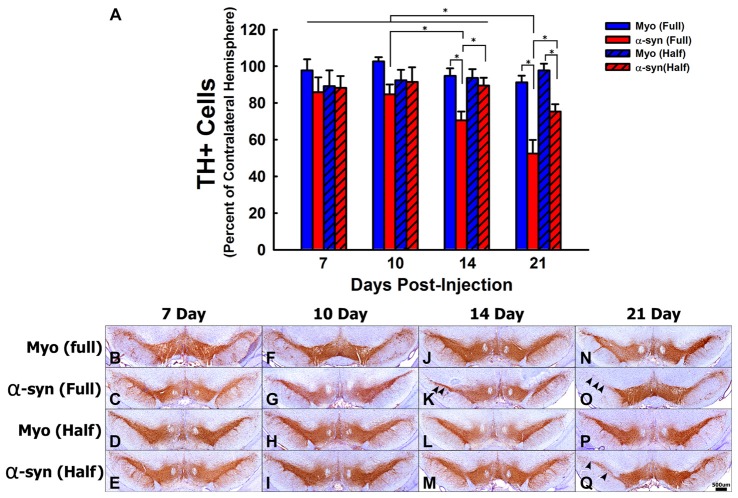
The time course and concentration response of tyrosine hydroxylase positive (TH+) cell loss following α-syn knockdown within nigrostriatal neurons. Rats received unilateral substantia nigra pars compacta (SNc) injections of AAV2/5 expressing an α-syn short hairpin RNA (shRNA) or a myocardin (Myo) control shRNA. AAV2/5 expressing the respective shRNAs was injected at a titer of 2.6 × 10^12^ vg/ml (Full) or 1.3 × 10^12^ vg/ml (Half). Rats were then sacrificed at 7, 10, 14, and 21 days post-surgery, and the number of TH+ neurons in the SNc was estimated using unbiased stereology. Columns in panel **(A)** represent mean number of TH+ cells in the injected SNc, expressed as the percent of TH+ cells in the contralateral SNc, +1 SEM (*n* = 6–7/group), in animals receiving Myo Full shRNA (solid blue columns), α-syn Full shRNA (solid red columns), Myo Half shRNA (hatched blue columns), or α-syn Half shRNA (hatched red columns). *Significantly different (ANOVA: *p* < 0.05). Representative images of TH positive neurons of the SNc for all shRNA concentrations and time points are shown panels **(B–Q)**. Arrowheads indicate areas in which TH+ cell loss was observed. Scale bar in **(Q)** represents 500 μm and applies to all other panels.

**Figure 2:**
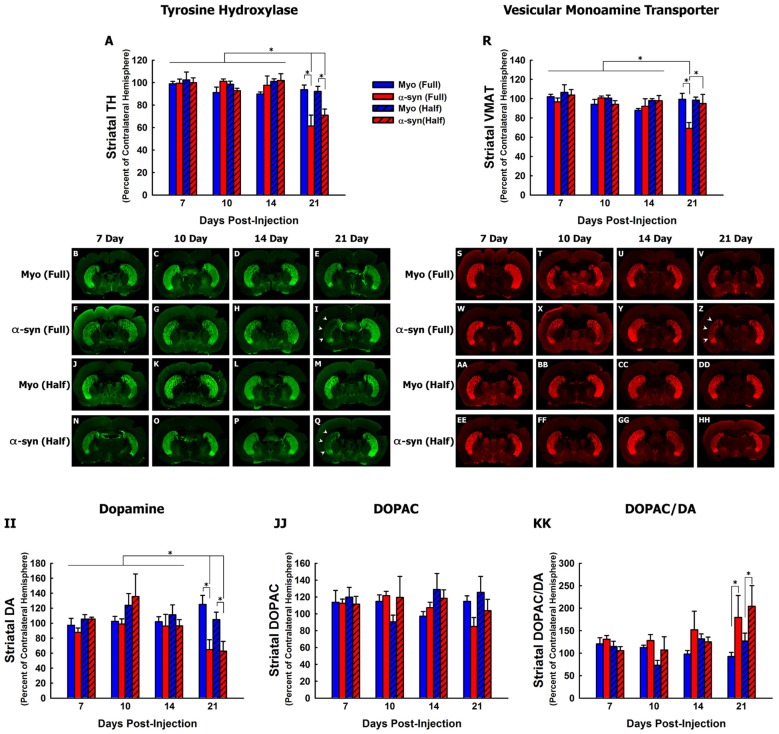
Time course and concentration response of nigrostriatal axon terminal degeneration following α-syn knockdown. Rats received unilateral injections of AAV2/5 expressing the α-syn shRNA or a myocardin control shRNA. AAV2/5 expressing the respective shRNAs was injected at a titer of 2.6 × 10^12^ vg/ml (Full) or 1.3 × 10^12^ vg/ml (Half). Rats were then sacrificed at 7, 10, 14 and 21 days post-surgery, and striatal TH, VMAT, dopamine (DA) and 3,4-dihydroxyphenylacetic acid (DOPAC) were quantified. Columns represent the mean total signal intensity of striatal TH **(A)** or VMAT **(R)**, or the mean concentration of striatal DA **(II)**, DOPC **(JJ)** or the DOPAC to DA ratio **(KK)**, expressed as the percent of the contralateral striatum, +1 SEM (*n* = 5–8/group), in animals receiving Myo Full shRNA (solid blue columns), α-syn Full shRNA (solid red columns), Myo Half shRNA (hatched blue columns), or α-syn Half shRNA (hatched red columns). *Significantly different (ANOVA, *p* < 0.05). Representative images of TH+ **(B–Q)** and VMAT+ **(S–HH)** striata are shown. Arrowheads in panels **(I,Q,Z)** indicate areas in which nigrostriatal terminal loss was observed.

**Figure 3:**
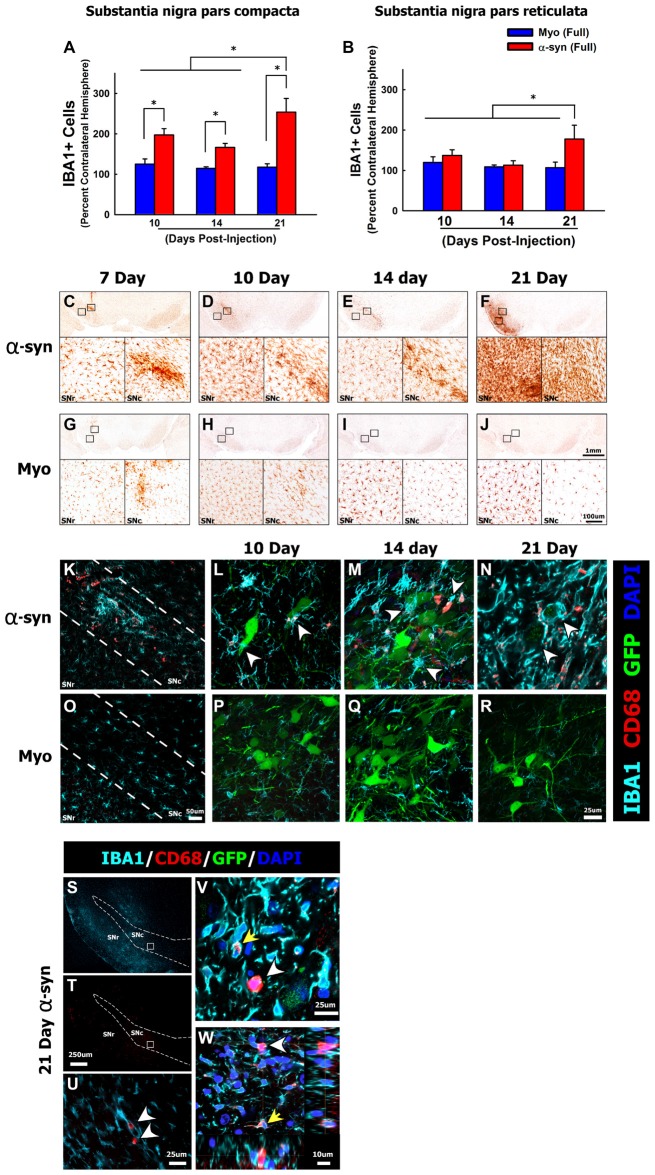
α-syn knockdown increases reactive microglia in the SNc. Rats received a single injection of AAV2/5 (2.6 × 10^12^ vg/ml; Full) expressing a target α-syn shRNA or a myocardin control shRNA. Rats were then sacrificed at 7, 10, 14 and 21 days post-surgery, the number of IBA1+ microglia were quantified using unbiased stereology. Columns in panel **(A,B)** represent mean number of IBA1+ microglia in the ipsilateral SNc and substantia nigra pars reticulata (SNr), respectively, expressed as the percent of IBA1+ microglia in the contralateral SNc and SNr, +1SEM (*n* = 5–8/group), in animals receiving Myo Full shRNA (solid blue columns), α-syn Full shRNA (solid red columns). Representative images show low and high magnification images of IBA1+ microglia within the SNc and SNr in animals treated with the α-syn shRNA **(C–F)** or myocardin shRNA **(G–J)**. Panel **(K)** depicts CD68+ (red), IBA1+ (teal) microglia within the anatomical boundaries of the SNc 10-days following injection of the α-syn shRNA. Arrowheads in panels **(L–N)** depict CD68+ (red), IBA1+ (teal) reactive microglia surrounding α-syn shRNA transduced neurons (indicated by green fluorescent protein (GFP) reporter; green) at 10- **(L)**, 14- **(M)**, and 21-days **(N)** post-injection. Panels **(O–R)** depicts IBA1+ microglia that are largely devoid of CD68 immunoreactivity within the anatomical boundaries of the SNc at 10 **(O,P)**, 14 **(Q)** and 21 days **(R)** following injection of α-myocardin shRNA. Numerous round CD68+ cells, which expressed little to no IBA1, were found throughout the entire ventral midbrain 21-days post-α-syn-shRNA expression **(S,T)**. Panel **(U)** corresponds to area within the box in panels **(S,T)**. The yellow arrow in panels **(V,W)** depicts the normal punctate CD68 immunoreactivity that was observed within IBA1+ microglia. The white arrowheads in panels **(V,W)** depict ameboid cells expressing high levels of CD68 with little to no colocalization with IBA1. *Significantly different (ANOVA: *P* < 0.05). The scale bars in panel **(J)** and the inset in panel **(J)** represents 1 mm and 100 μm respectively, and apply to the corresponding panels and insets in **(C–I)**. The scale bar in panel **(O)** represents 50 μm and applies to panel **(K)**. The scale bar in panel **(R)** represents 25 μm and apply to panels **(L,M**,**P,Q)**. Scale bar in panel **(T)** represents 250 μm and applies to panel **(S)**. Scale bar in panels **(U,V)** represent 25 μm. Scale bar in Panel **(W)** represents 10 μm.

**Figure 4:**
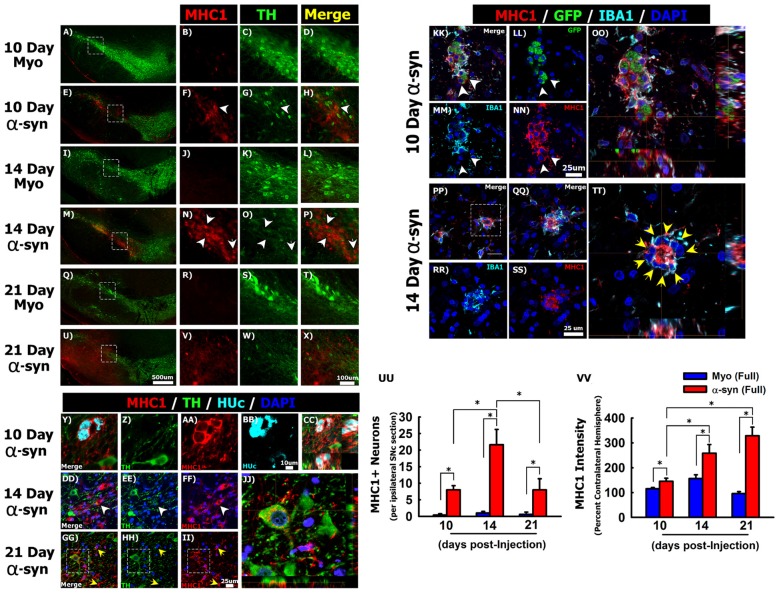
Silencing α-syn in nigral neurons induces major histocompatibility complex class 1 (MHC-1) expression. Rats received a single injection of AAV2/5 (2.6 × 10^12^ vg/ml; Full) expressing a target α-syn shRNA or a myocardin control shRNA. Rats were then sacrificed at 10, 14, and 21 days post-surgery. Panels **(A–X)** show MHC-1 (red) immunoreactivity near TH (green) neurons in myocardin shRNA treated animals at 10 days **(A–D)**, 14 days **(I–L)** and 21 days **(Q–T)** post injections. MHC-1 expression in α-syn shRNA treated animals at 10 days **(E–H)**, 14 days **(M–P)** and 21 days **(U–X)** post injection. White arrowheads in **(F–H)** and **(N–P)** indicate neuronal MHC-1 expression. Panels **(Y–CC)** show co-localization of MHC-1 (red) with the neuronal marker HUc (teal) within the SNc 10 days following α-syn shRNA injection. Panels **(DD–JJ)** show co-localization of MHC-1 expression in TH+ neurons of the α-syn shRNA injected SNc. White arrowheads in **(DD–FF)** indicate MHC-1 expression in a neuron that also expresses low levels of TH. Yellow arrows in **(GG–II)** indicate MHC-1 expressing microglia. Panel **(JJ)** shows a composite z-stack image acquired using confocal microscopy, corresponding to the area within the box in panels **(GG–II)** showing MHC-1 expression on the surface of a TH+ neuron. Panels **(KK–TT)** show representative micrographs of transduced neurons (GFP+; green) within the α-syn shRNA injected SNc stained for IBA1 (teal) and MHC-1 (red). White arrowheads in **(KK–NN)** indicate cell surface expression of MHC-1 on GFP+ transduced neurons, which does not co-localize with the microglial marker IBA1 **(OO)**. Yellow arrows in **(TT)** indicate IBA1+ microglia surrounding a neuron with high MHC-1 expression. Panels **(QQ–TT)** correspond to the area within the box in **(PP)**. Panels **(OO)** and **(TT)** are composite z-stack images acquired using confocal microscopy. Columns in **(UU)** represent the mean number of MHC-1+ neurons, + 1 SEM (*n* = 4–5/group), counted from four sections of the ipsilateral ventral midbrain, spanning the rostral-caudal axis of the SNc. Columns in **(VV)** represent the mean MHC-1 fluorescent intensity within ventral midbrain of the ipsilateral hemisphere, expressed as a percent of the mean MHC-1 fluorescent intensity within ventral midbrain of contralateral hemisphere, +1 SEM (*n* = 4–5/group). *Significantly different (ANOVA: *P* < 0.05). Scale bar in **(U)** represents 500 μm and applies to panels **(A**,**E,I,M,Q)**. Scale bar in **(X)** represents 100 μm and applies to **(B–D**,**F–H**,**J–L**,**N–P**,**R–T,V,W)**. Scale bar in panel **(BB)** represents 10 μm and applies to panels **(Y–AA)**. Scale bar in **(II)** represents 25 μm and applies to panels **(DD–HH)**. Scale bars in **(NN)** and **(SS)** represent 25 μm and apply to panels **(KK–MM**,**PP–RR)**.

### Fluorescence Intensity Analysis

Tissue was processed for immunofluorescent detection (as described above) of TH and α-syn. Images were acquired using a Nikon A1 laser scanning confocal system with standardized laser and detection settings that were optimized to avoid saturation. Regions of interest were drawn around approximately 100–150 individual TH+ neurons, per hemisphere, per animal. a priori criteria were set such that all cells must be TH+, have a distinctive cell boundary, and a clearly discernable nucleus to be included in the analysis. To detect sub-regional differences in cellular TH, midbrain DA neurons were subdivided into the medial ventral tegmental area, the lateral VTA, dorsal tier SNc, ventral tier SNc, and lateral SNc (see Supplementary Figure 5B). Outlined cells were treated as replicates that were averaged within a single animal (or within the respective midbrain subregion), and the mean fluorescence intensity was calculated for each hemisphere and expressed as the percent of the mean fluorescent intensity of the contralateral hemisphere (or the respective sub-region of the contralateral hemisphere).

To quantify the fluorescence intensity of MHC-1, tissue was processed for immunofluorescent detection (as described above) of rat MHC-1. High magnification images of the ventral midbrain were obtained from four sections spanning the rostral-caudal axis of the SNc, within the ipsilateral and contralateral hemispheres. Confocal images were acquired as described above. Nikon NIS elements software was used to perform automated-unbiased pixel fluorescence intensity analysis. The mean background fluorescence intensity in the 594 channel was measured in a subset of animals representing each experimental group to be analyzed. This mean background fluorescence intensity value was set as the lower threshold for automated fluorescence intensity analysis and was applied to all samples analyzed. The automated measurement tool in NIS elements was used to quantify the mean fluorescence intensity for all pixels above the preset, lower threshold. The mean MHC-1 fluorescence intensity was averaged over all sections within animal, and expressed as a percent of the mean fluorescence intensity of the contralateral hemisphere.

### RNAscope *in Situ* Hybridization

RNAscope *in situ* hybridization for the promoter within the AAV genome was combined with immunohistochemical detection of IBA1+ microglia. Forty micrometer thick tissue was processed for RNAscope detection of the promoter within the viral genome using custom VS probes according to the manufacturer’s instructions (Advanced Cell Diagnostics, Hayward, CA, USA) and previously published methods (Polinski et al., 2016). RNAscope was developed with 3-3′-diaminobenzidine tetrahydrochloride, after which tissue was counterstained for IBA1 using the immunohistochemical procedures detailed above, with the exception that the Vector SG reagent (Vector Laboratories) was used as the chromagen (Polinski et al., 2016).

### Statistical Analysis

Power analyses were conducted to determine optimal sample size required to detect a statistical difference at *p* < 0.05 with a power of 0.8. *A priori* exclusion criteria stated that animals displaying a complete absence of transduction (no GFP reporter gene expression) were excluded from analysis. The experimenter was blind to all experimental conditions during data collection and analysis. One-way analysis of variance (ANOVA) tests were used to detect statistical significance between two or more groups containing a single independent variable. Repeated measure two-way ANOVAs were used to detect statistical significance between two or more groups when there were two independent variables in the experiment. A repeated measures ANOVA was used to detect subregional differences within subjects. A *p* value of less than or equal to 0.05 was considered statistically significant. If the ANOVA revealed an interaction of statistical significance Tukey’s test was used for multiple comparisons among groups.

### Data Availability

The raw data supporting the author’s conclusions will be made available by the authors, without undue reservation, to any qualified researcher.

## Results

### Specificity of α-syn shRNA-Induced Nigrostriatal Toxicity

We first sought to unequivocally confirm that nigrostriatal cell loss observed following α-syn knockdown was not due to non-specific, or off-target, RNA interference (RNAi) toxicity. The synuclein family consists of α, β and γ synuclein, three proteins which share sequence homology in the amino terminus. γ-synuclein is expressed in the brain and periphery but is the least conserved of the synucleins (George, 2001); however, α- and β-synuclein (β-syn) are largely co-expressed in the central nervous system and they appear to be functionally redundant (Chandra et al., 2004; Greten-Harrison et al., 2010). Although our α-syn shRNA specifically targets a position of the α-syn transcript that does not share sequence homology with either β- or γ-synuclein (Supplementary Figure 1A), we aimed to ensure that the α-syn shRNA did not affect expression of β-syn, as this could mask the true effects of α-syn silencing (as has been observed with several α-syn germline knockout mice (Abeliovich et al., 2000; Chandra et al., 2004; Greten-Harrison et al., 2010)). PC12 cells were transduced with LV expressing either the α-syn shRNA or a scrambled control shRNA, and α- and β-syn protein levels were quantified 5 days post-transduction. The α-syn shRNA significantly reduced α-syn protein (*t*-test; *t*_(10)_ = 2.37, *p* = 0.039), however there was no change in β-syn protein (Supplementary Figures 1B,C).

Next we examined the consequences of α-syn shRNA administration in α-syn germline KO and WT mice. α-syn KO or WT mice received unilateral SNc injections of AAV expressing the α-syn shRNA or a scrambled control shRNA and TH positive (TH+) nigral neuron numbers were quantified 1-month later. Expression of the α-syn shRNA significantly decreased TH+ nigral neurons in WT mice (~45% reduction; Supplementary Figures 1D,E; ANOVA: *F*_(3,24)_ = 4.465, *p* < 0.05), but had no effect on TH+ neurons in α-syn KO mice (Supplementary Figures 1D,F). These data provide further evidence that loss of endogenous α-syn is toxic to nigrostriatal neurons, and combined with previously published controls (Gorbatyuk et al., 2010), exclude the possibility that nigrostriatal cell loss following α-syn shRNA administration is due to non-specific RNAi-induced toxicity or off-target activity.

### Time Course of Nigrostriatal Pathology Following α-syn Knockdown

Two separate titers of AAV 2/5 expressing an shRNA targeting endogenous rat α-syn were unilaterally injected into the SNc of adult rats, and nigrostriatal pathology was examined at 7, 10, 14 and 21 days post-surgery. We observed successful transduction of TH+ cells in the SNc at all time points examined (indicated by the GFP transduction marker), however, GFP transgene expression increased over the time course (Supplementary Figure 2 shows GFP in myocardin shRNA treated animals). In animals receiving the α-syn shRNA, expression of the GFP reporter gene seemed to decrease over time (data not shown), likely the result of ongoing pathology within α-syn shRNA expressing neurons.

The level of α-syn knockdown was analyzed using confocal microscopy to quantify the intensity of α-syn fluorescence within TH+ nigrostriatal soma (Supplementary Figure 3). Prior to this analysis, WT and α-syn KO brain were stained using the α-syn antibody (clone 42/α-synuclein) to confirm the specificity of the antibody. No signal was observed in α-syn KO brains (Supplementary Figures 3A,B). Further, immunoblotting of recombinant monomeric α-syn protein or striatal lysates from WT mice, α-syn KO mice or rat brain yielded a single band resolving at correct molecular weight (Supplementary Figure 3C).

There was no change in the fluorescence intensity of α-syn at any time point following injections of either the FULL or HALF titer AAV-myocardin-shRNA, thus solely 21-days post myocardin shRNA is shown (Supplementary Figures 3D–G,X–AA,RR). The α-syn shRNA (FULL) significantly reduced α-syn immunoreactivity by 44%, 48%, 50% and 57% as compared to the contralateral hemisphere at 7, 10, 14 and 21 days post-injection, respectively (Supplementary Figure 3RR; ANOVA: *F*_(7,18)_ = 16.865, *p* < 0.0001). The α-syn shRNA (HALF), significantly reduced α-syn immunoreactivity by 24%, 25%, 40% and 47% as compared to the contralateral hemisphere at 7, 10, 14, and 21 days-injection, respectively (Supplementary Figure 3RR; ANOVA: *F*_(7,20)_ = 8.092, *p* < 0.0001).

Next the time course of TH+ cell loss in the SNc following α-syn knockdown was examined (Figures 1A–Q). There was no change in TH+ neuron numbers at any time following expression the myocardin shRNAs. The α-syn shRNA significantly decreased TH+ cell numbers within the injected SNc (ANOVA: main effect of treatment *F*_(3,82)_ = 12.36, *p* < 0.0001), and this cell loss progressed over time (ANOVA: main effect of time *F*_(3,82)_ = 4.253, *p* < 0.007). The α-syn shRNA (Half) significantly decreased TH+ neurons in the injected SNc at 21 days post-injection (~25% loss; Figures 1A,Q). The α-syn shRNA (Full) produced a progressive loss of TH+ cells in the SNc beginning at 14 days (~30% loss; Figures 1A,K) and continuing to 21 days post-injection (~50% loss; Figures 1A,O). Further, administration of α-syn shRNA (Full) resulted in a more pronounced loss of TH+ nigrostriatal neurons as compared to the α-syn shRNA (Half) at the 21-day time point, demonstrating a time and concentration dependent loss of TH+ neurons following knockdown of endogenous α-syn. To determine if TH+ cell loss reflects actual nigral cell loss or merely a phenotypic loss of TH expression, we quantified total neurons within the SNc using the pan-neuronal marker HUc (Supplementary Figure 4). The total number of HUc+ neurons within the ipsilateral SNc was significantly reduced by ~31% and ~36% 21-days following delivery of α-syn shRNA (Half) and α-syn shRNA (Full), respectively (ANOVA: *F*_(4,24)_ = 3.61, *p* < 0.01). There was also a non-significant reduction in HUc+ neurons 14 days following delivery of the α-syn shRNA (Full; ~27%).

Manipulation of α-syn alters TH expression (Baptista et al., 2003; Yu et al., 2004; Alerte et al., 2008), thus, we also analyzed TH fluorescence intensity within individual nigral neurons to probe for changes in TH expression (Supplementary Figures 5A–C). In an effort to detect any sub-regional differences in cellular TH intensity, we subdivided midbrain DA neurons into the medial ventral tegmental area (VTA-Medial), the lateral VTA (VTA-Lateral), the dorsal tier SNc (SNc-Dorsal), the ventral tier SNc (SNc-Ventral), and the lateral SNc (SNc-Lateral; Supplementary Figures 5A,B). There was no change in TH fluorescence intensity among the myocardin shRNA treated animals (data not shown), thus, data from these animals is presented as a single time point and treated as time zero after α-syn shRNA administration (Supplementary Figure 5C). There was no change in TH fluorescence intensity within VTA neurons in any groups analyzed. However, α-syn shRNA expression significantly decreased TH fluorescence intensity within nigral neurons over time (ANOVA: *F*_(4,16)_ = 14.675, *p* < 0.0001). Further, there was a significant interaction between time after α-syn shRNA treatment and the midbrain dopaminergic sub-region, in which TH fluorescence intensity in the ventral tier of the SNc was significantly lower than the VTA as well as the other sub-regions of the injected SNc at the 21-day time point (Supplementary Figure 5C; ANOVA: *F*_(16,40)_ = 2.675, *p* < 0.01).

We next analyzed nigrostriatal axon terminal integrity by quantifying levels of striatal TH (Figures 2A–Q) and VMAT (Figures 2R–HH) protein, and striatal concentrations of DA and DOPAC (Figures 2II–KK). The α-syn shRNA (Full) and α-syn shRNA (Half) significantly decreased TH+ fibers within the striatum 21-days post-injection (Figures 2A,I,Q; ANOVA: main of effect of time *F*_(3,82)_ = 15.01, *p* < 0.0001; interaction between shRNA treatment and time *F*_(9,82)_ = 3.467, *p* < 0.001). In contrast, only the α-syn shRNA (Full) decreased VMAT+ nigrostriatal fibers 21-day post-injection (Figures 2R,Z; ANOVA main effect of α-syn shRNA treatment *F*_(3,82)_ = 3.214, *p* < 0.02; main effect of time *F*_(3,83)_ = 3.695, *p* < 0.01).

Striatal DA concentrations were significantly reduced 21-days following injection the α-syn shRNA (Full) and α-syn shRNA (Half; Figure 2II; ANOVA: main effect of treatment *F*_(3,94)_ = 2.716, *p* < 0.04; main effect of time *F*_(3,94)_ = 4.957, *p* < 0.003; interaction effect between treatment and time *F*_(9,94)_ = 2.814, *p* < 0.005). There was no change in striatal DOPAC concentrations regardless of treatment or time point (Figure 2JJ). However, there was a significant increase in the DOPAC to DA ratio at 21 days following injection of the α-syn shRNA (Full) and α-syn shRNA (Half; Figure 2KK; ANOVA main effect of treatment *F*_(3,94)_ = 3.091, *p* < 0.03; main effect of time *F*_(3,94)_ = 3.463, *p* < 0.01).

### Neuroinflammatory Response Initiated by α-syn Knockdown in Nigrostriatal Neurons

After characterizing the time line of pathology, we sought to determine if α-syn knockdown within nigral neurons elicits an inflammatory response. Here we focused on the α-syn shRNA (FULL) as this titer resulted in demonstrable neuronal dysfunction throughout the time course. In all rats we observed a moderate, and roughly equal, amount of gliosis along the needle track and the injection site at the 7-day time point (Figures 3C,G). This gliosis is an artifact of surgery, thus the 7-day time point was not included in additional analyses. The α-syn shRNA (Full) significantly increased the number of IBA1+ microglia within the injected ventral midbrain, beginning at 10 and 14 days post-injection, and increasing further at 21 days post-injection (ANOVA: main effect of treatment *F*_(5,30)_ = 9.098, *p* < 0.0001; Figures 3A,D–F). In addition, by 21 days post-injection the number of IBA1+ microglia was significantly increased in the substantia nigra pars reticulata (SNr; Figures 3B,F; ANOVA: main effect of treatment *F*_(5,31)_ = 2.608, *p* < 0.0442). Beyond microglia lining the needle track and injection site at early time points, microglial numbers did not change following injection of the myocardin shRNA (Figures 3A,B,G–J). Interestingly, at several time points post-α-syn shRNA administration the abundance of IBA1+ microglia appeared, from a purely qualitative point of view, to be increased far beyond the increase in the number of IBA+ microglia that were objectively quantified (e.g., Figure 3B vs. Figures 3F,J). This appears to result from an increase in the expression of IBA1 per cell, which exceeds the increase in actual IBA1+ cell numbers. Beginning at the 10-day time point, microglia in the α-syn shRNA injected SNc were reactive and phagocytic as indicated by increased CD68 immunoreactivity (Figure 3K). CD68+ microglia were in close apposition to GFP+ transduced neurons, and appeared to be extending processes to surround and engulf transduced neurons, indicative of neuronophagia of living neurons (arrowheads in Figures 3L–N). Further at 21 days post-α-syn-shRNA injection, we observed numerous ameboid CD68+ cells throughout the ventral midbrain (Figures 3S–U). The ameboid cells were in close apposition to IBA1+ microglia but displayed little to no IBA1 expression themselves (Figures 3V,W), indicating that they may be either ameboid microglia or infiltrating mononuclear cells. By comparison the microglia within the brains of myocardin shRNA treated animals displayed very low CD68 immunoreactivity (Figures 3O–R).

Mesencephalic DA neurons increase MHC-1 expression in the parkinsonian brain, and MHC-1 expression in cultured neurons results in the recruitment of immune cells and subsequent death of the affected neuron (Cebrián et al., 2014b). Thus, we next sought to determine if α-syn knockdown induces MHC-1 expression in nigral neurons. We observed either very weak, or a complete absence of MHC-1 immunoreactivity in the SNc of animals injected with the myocardin shRNA (Figures 4A–D,I–L,Q–T). In contrast, knockdown of α-syn increased MHC-1 expression within the SNc. Most of the cells expressing MHC-1 between 10 days (Figures 4E–H) and 14 days (Figures 4M–P) post-α-syn shRNA injection did not express TH, but resembled nigral neurons in terms of their anatomical location, size, and morphology. At the 10 and 14-day time points, all MHC-1+ neurons were almost exclusively found within the ventral tier of the SNc (Figures 4E,M), which is the location where we observed the most pronounced cell loss at the 21 day time point. At the 21-day time point MHC-1 immunoreactivity was no longer confined to the SNc but was observed throughout the ventral midbrain of the injected hemisphere. Further, at 21 days post-α-syn shRNA, MHC-1+ cells no longer presented a neuronal morphology, instead MHC-1 staining appeared diffuse or found on cells morphologically resembling microglia throughout the ventral midbrain (Figures 4U–X).

To confirm that MHC-1 expression was occurring on nigral neurons in the SNc, we analyzed colocalization of MHC-1, TH, and the pan-neuronal marker HUc (Figures 4Y–JJ). At the 10-day time point MHC-1 was expressed on HUc+ neurons that were TH- (Figures 4Y–CC). At the 14-day time point MHC-1 was expressed on HUc+ neurons (data not shown), in addition to neurons with limited TH expression (arrowheads in Figures 4DD–FF). Finally, at the 21-day time point, many of the remaining TH+ neurons in the injected SNc demonstrated cell surface MHC-1 expression (Figures 4GG–JJ).

Microglia also express MHC-1, and many MHC-1+ cells with distinct microglial morphology were in the α-syn shRNA injected SNc 21 days post-injection (yellow arrows Figures 4GG–II). As such, we wanted to confirm that MHC-1 expression was not solely an artifact of microglia surrounding transduced neurons. Ten days post α-syn shRNA injection, GFP+ transduced neurons displayed robust cell surface MHC-1 expression (Figures 4KK–OO). This MHC-1 expression distinctly surrounded the soma of GFP+ neurons, and although there were also IBA1+ microglia surrounding the same neurons, the IBA1 and MHC-1 signals rarely co-localized (Figure 4OO). At 14 days post α-syn shRNA injection, neuronal MHC-1 expression was more robust, filling the soma in addition to cell surface expression (Figure 4PP). Further, at the 14-day time point MHC-1+ neurons were often completely surrounded by IBA1+ microglia (Figures 4PP–TT), again indicative of neuronophagia.

We next quantified the number of MHC-1 expressing neurons within the ventral midbrain between 10 days and 21 days post-injection. MHC-1+ neurons were extremely rare or not present in the contralateral hemisphere, thus the contralateral hemisphere could not be used as an internal control in the analysis. Within the ipsilateral hemisphere, there was a significant increase in MHC-1+ neurons at 10, 14 and 21 days following injection of the α-syn shRNA (Figure 4UU; ANOVA: *F*_(5,18)_ = 11.754, *p* < 0.0001).

We also quantified MHC-1 fluorescence intensity within the ventral midbrain to detect changes in MHC-1 expression in all cell types, including microglia (Figure 4VV). The α-syn shRNA progressively increased MHC-1 fluorescence intensity from 10-days post-injection to peak at 21-days post-injection (Figure 4VV; ANOVA: *F*_(5,18)_ = 48.536, *P* < 0.0001). These collective data show that α-syn knockdown in nigral neurons induces MHC-1 expression in transduced neurons, prior to any neuronal loss, which coincides with the recruitment of reactive microglial to MHC-1 expressing neurons.

**Figure 5:**
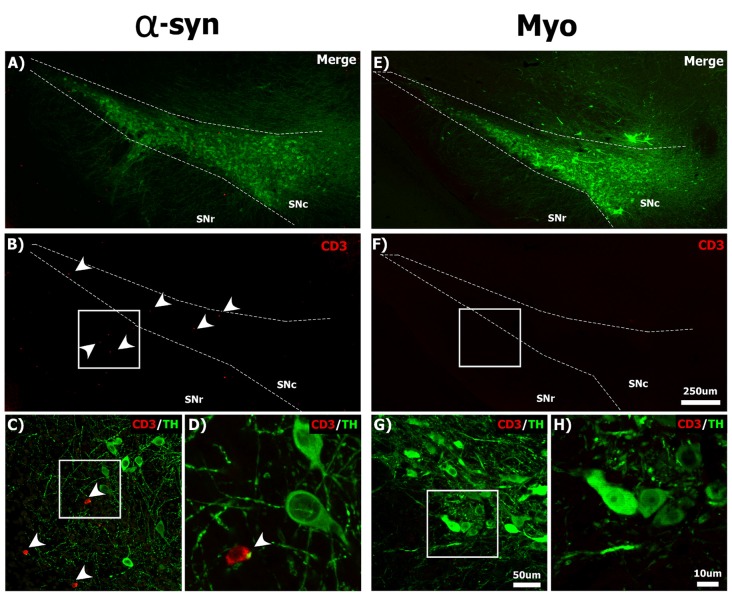
Silencing α-syn in nigral neurons results in the infiltration of CD3+ T-cells to the SNc. Rats received a single injection of AAV2/5 (2.6 × 10^12^ vg/ml; Full) expressing the α-syn or myocardin shRNA and were sacrificed 21 days post-surgery. Panels show CD3+ (red) T-cells near TH+ (green) nigral neurons in the SNc of α-syn shRNA **(A–D)** and myocardin shRNA **(E–H)** treated animals. White arrowheads in panels **(B–D)** indicate CD3+ T-cells. Scale bar in panel **(F)** represents 250 μm and applies to panel **(A,B,E)**. Scale bar in panel **(G)** represents 50 μm and applies to panel **(C)**. Scale bars in panels **(H)** represents 10 μm and applies to panel **(D)**.

Neuronal MHC-1 expression is associated with the recruitment of T-cells in the parkinsonian brain (Cebrián et al., 2014b), thus we next investigated whether α-syn knockdown caused infiltration of T-cells to the brain. We observed a number of CD3 + T-cells within the injected SNc, solely at 21-days post-α-syn shRNA administration, many of which were in close apposition to TH+ neurons (Figures 5A–D). In contrast we could not detect any CD3+ T-cells in the brains of animals receiving the myocardin shRNA (Figures 5E–H), or at other time points following α-syn knockdown (data not shown).

Neuronophagia activates programmed cell death within affected cells. Thus we next analyzed the SNc for levels of cleaved caspase-3, a marker of apoptosis (Supplementary Figure 6). Within the myocardin shRNA treated animals there was little to no cleaved caspase-3 immunoreactivity (Supplementary Figures 6A–C,G–I). There was sparse cleaved caspase-3 immunoreactivity within a limited number of cells 14-days following α-syn shRNA expression (Supplementary Figures 6D–F), however, by the 21-day time point, punctate cleaved caspase-3 immunoreactivity within the cytosol and nucleus of cells was prevalent. Most cells displaying strong cleaved caspase-3 immunoreactivity morphologically resembled neurons, and were TH-, though there was some sparse cleaved caspase-3 immunoreactivity in TH+ neurons as well (Supplementary Figures 6J–L).

Microglial from α-syn KO mice display a reactive phenotype (Austin et al., 2006, 2011). Thus, it is possible that loss of endogenous α-syn within microglia of the SNc (following transduction with AAV expressing the α-syn shRNA) results in increased cytokine secretion, which induces nigral neurons to express MHC-1 (Cebrián et al., 2014b). To investigate this possibility, we performed a combination of RNAscope *in situ* hybridization for the promoter of the AAV genome, and IBA1 immunohistochemistry to visualize microglia, to determine if any viral genomes were located within microglia. Although there was abundant punctate signal from the AAV genome within the transduced SNc, we were unable to detect any AAV genomes within IBA1+ microglia (Supplementary Figures 7A–C), supporting previous reports that AAV5 does not transduce microglia (Rosario et al., 2016). These data support the conclusion that loss of endogenous α-syn within nigral neurons is able to stimulate a neuron-initiated inflammatory cascade without directly transducing- or manipulating levels of α-syn within microglia.

### The Effects of α-syn Knockdown in Non-dopaminergic Neurons

**Figure 6:**
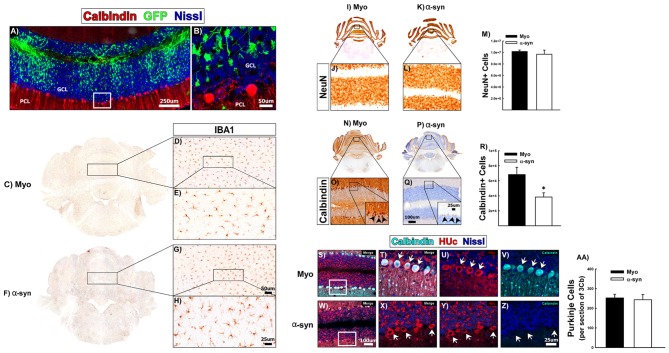
The effects of α-syn knockdown within purkinje cells of the cerebellum. Rats received a single injection of AAV2/5 (2.6 × 10^12^ vg/ml; Full) expressing an α-syn shRNA or a myocardin control shRNA. One-month post-surgery rats were sacrificed. Panels **(A,B)** show transduction within the granule cell layer (GCL) of the cerebellum, directly adjacent to the purkinje cell layer (PCL). High magnification of the area within the box in panel **(A)** is shown in panel **(B)**. Panels **(C–H)** shows IBA1+ microglia in Myo **(C–E)** and α-syn shRNA **(F–H)** treated animals. Unbiased stereology was used to quantify NeuN+ and calbindin+ neurons in the cerebellum. Columns in Panel **(M)** represent the mean number of NeuN+ cells from animals treated with the myocardin shRNA (black bars) or the α-syn shRNA (white bars). Representative images of NeuN+ cells of the cerebellum are shown for myocardin shRNA **(I,J)** and α-syn shRNA **(K,L)** treated animals. Columns in **(R)** represent mean number of calbindin+ cells within the cerebellum from animals injected with the myocardin shRNA (black bars) or the α-syn shRNA (white bars). Representative images of calbindin immunoreactivity within purkinje cells of the cerebellum from animals injected with the myocardin shRNA **(N,O)** and α-syn shRNA **(P,Q)** are shown. Error bars in **(M,R)** are + 1 SEM (*n* = 7–8/group). *Significantly different than myocardin treated group (*t*-test: *P* < 0.05). Panels **(S–Z)** show co-localization of calbindin (teal) with the pan-neuronal marker HUc (red) in the PCL of animals treated with the myocardin shRNA **(S–V)** or the α-syn shRNA **(W–Z)**. High magnification images of the boxes in panels **(S,W)** are shown in panels **(T–V)** and **(X–Z)**, respectively. Arrows indicate purkinje cells that are calbindin positive **(T–V)** or calbindin negative **(X–Z)**. Columns in panel **(AA)** represent the mean number of purkinje cells in the third lobule of the cerebellar vermis (3Cb) from animals treated with the myocardin shRNA (black bars) or the α-syn shRNA (white bars). Scale bars in panels **(A,B)** represent 250 μm and 50 μm respectively. Scale bar in panels **(G)** represents 50 μm and applies to panel **(D)**. Scale bar in panel **(H)** represents 25 μm and applies to panel **(E)**. Scale bar in panel **(Q)** represents 100 μm and applies to panels **(J,L,O)**. Scale bar in the inset in panel **(Q)** represents 25 μm and applies to the inset in panel **(O)**. Scale bar in panel **(W)** represents 100 μm and applies to panel **(S)**. Scale bar in panel **(Z)** represents 25 μm and applies to panels **(T–V)** and **(X,Y)**.

To determine if α-syn knockdown toxicity and the corresponding neuroinflammatory sequela are specific to DA neurons, we analyzed the consequences of α-syn knockdown in a non-dopaminergic neuronal subpopulation. We choose to investigate the effects of α-syn knockdown within the granule cell layer (GCL) of the cerebellum, as neurons in the rat cerebellum expresses high levels of α-syn, and granule cells predominantly utilize glutamate as a neurotransmitter (Wersinger et al., 2004). One-month post-surgery there was abundant transduction that was largely confined to the GCL (Figures 6A,B). There was no change in IBA1+ microglia within the cerebellum following α-syn knockdown (Figures 6C–H). There was no change in the total number of NeuN+ neurons within the cerebellum following α-syn knockdown (Figures 6I–M). Purkinje cells to do not express NeuN (Mullen et al., 1992), thus, we also processed cerebellar tissue for immunohistochemical detection of the common purkinje marker, calbindin D-28K (calbindin), to probe the integrity of purkinje neurons (Figures 6N–R). α-syn knockdown resulted in a striking loss of calbindin+ purkinje cells (Figure 6R; ~50% loss; *t*-test: *t*_(13)_ = −2.738, *p* = 0.016). However, while performing calbindin+ cell counts, there were many remaining purkinje cells, which had very weak calbindin-immunoreactivity (arrows in the inset in Figure 6Q). This suggested that α-syn knockdown was not necessarily resulting in death of purkinje cells, but merely a phenotypic loss of calbindin expression.

To determine if calbindin negative purkinje cells were still present, we analyzed the PCL for expression of neuronal marker, HUc. Figures 6S–V shows colocalization of HUc and calbindin within purkinje cells of myocardin shRNA treated animals (arrows in Figures 6T–V). Within the α-syn shRNA injected cerebellum (Figures 6W–Z), calbindin immunoreactivity was almost completely absent, however, there remained strong HUc expression within cells of the purkinje layer (arrows in Figures 6X–Z). Indeed there was no change in the number of purkinje cells in the lobule 3 of the cerebellar vermis (an area immediately adjacent to the injection site) of α-syn shRNA treated animals (Figure 6AA). These data demonstrate that α-syn knockdown in cerebellar granule cells causes a phenotypic loss of calbindin expression within neighboring, non-transduced purkinje cells, suggestive of a functional deficit in the former. Importantly, our data show that α-syn knockdown does not cause neurodegeneration of the non-dopaminergic neurons of the cerebellum.

## Discussion

### Mechanistic Model of α-syn shRNA-Induced Toxicity

**Figure 7:**
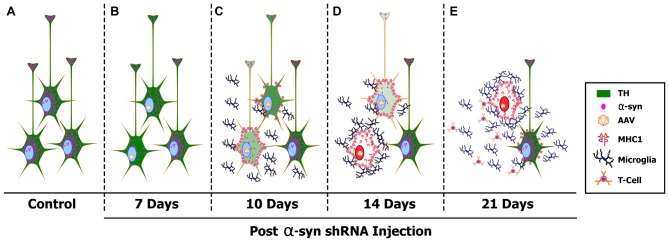
A proposed mechanism of α-syn shRNA induced toxicity. Healthy, TH+ (green) nigral neurons express high levels of α-syn (pink dots) **(A)**. Delivery of adeno-associated virus (AAV) expressing an shRNA targeting endogenous α-syn results in a rapid knockdown of α-syn expression that was detected as early as 7 days post-delivery **(B)**. Silencing endogenous α-syn results in a progressive decrease in TH expression within individual nigral neurons, as well as an overall loss of TH+ neurons within the SNc. Coinciding with loss of TH expression, nigral neurons up-regulate cell surface expression of MHC-1 as early as 10 days post-injection **(C)**. MHC-1 up-regulation results in recruitment of reactive microglia and the infiltration of CD3+ T-cells to the injected SNc. Recruited immune cell then surround and engulf MHC-1+ neurons, culminating in the death of nigral neurons *via* the induction of apoptotic cell death (red nucleus), and a corresponding loss of nigrostriatal terminals **(D,E)**. Cytokines released from reactive microglia in the affected SNc cause neighboring, non-transduced nigral neurons to increase surface expression of MHC-1, creating a cycle of toxicity **(E)**.

Here we have characterized the early pathological events that occur following α-syn knockdown in mature nigral neurons. Based on the data provided we propose a mechanistic model of toxicity elicited by α-syn silencing (Figure 7). Expression of the α-syn shRNA reduces α-syn protein as early as 7 days (Figure 7B), resulting in a progressive decrease in TH expression within nigral neurons, and an overall loss of nigral neurons over the 21-day time course. Nigral neurons up-regulate MHC-1 expression as early as 10-days following AAV-α-syn-shRNA injection, a time point prior to neurodegeneration (Figure 7C). MHC-1 up-regulation coincides with the recruitment of reactive microglia to affected neurons, followed by the infiltration of CD3+ T-cells to the injected midbrain. Recruited immune cells surround and engulf MHC-1+ neurons, culminating in the death of nigral neurons, and a corresponding loss of nigrostriatal terminals (Figures 7D,E). Cell death elicited by α-syn knockdown appears to be apoptotic and specific to dopaminergic neurons of the SNc, as neurodegeneration was not observed in the non-dopaminergic neurons of the cerebellum or in dopaminergic neurons of the VTA (Gorbatyuk et al., 2010). Finally, DA neurons in the ventral tier of the SNc were more sensitive to α-syn knockdown, as many of these metrics were exacerbated or observed solely in this population.

### The Critical Threshold of α-syn Knockdown

To date, the majority of knowledge on the biological function of α-syn, and more notably the pathological role of α-syn in Parkinson’s disease, has been gained by examining the consequences of increasing α-syn concentrations within a cellular environment (Chesselet, 2008). Although this approach has yielded a significant amount of information, an alternative though equally productive approach, is to examine the consequences of removing endogenous α-syn from the cell. This was initially attempted through the generation of several different lines of α-syn KO mice, however, despite modest changes in neurotransmission, germline removal of α-syn does not result in any major biological change or recapitulate Parkinson’s disease pathology (Abeliovich et al., 2000; Cabin et al., 2002, 2005; Dauer et al., 2002; Schlüter et al., 2003). This lack of an effect is likely the result of developmental genetic compensation (Perez et al., 2002; Schlüter et al., 2003; Chandra et al., 2004; Robertson et al., 2004; Kuhn et al., 2007; Wang et al., 2009; Ubhi et al., 2010; Cali et al., 2012; Benskey et al., 2016a; Ludtmann et al., 2016). For example, compared to WT mice, α-syn KO mice have 369 differentially expressed genes, including genes involved in apoptosis, neurotrophic signaling, and other, functionally redundant members of the synuclein family (Schlüter et al., 2003; Chandra et al., 2004; Robertson et al., 2004; Kuhn et al., 2007). As such, genetic compensation may mask the true consequences of loss of α-syn function, including the intra-neuronal sequela ultimately leading to an induction of a severe inflammatory response. Thus, many laboratories have begun examining the consequences of removing α-syn from mature neurons (Gorbatyuk et al., 2010; Khodr et al., 2011, 2014; Zharikov et al., 2015; Benskey et al., 2016a; Collier et al., 2016).

Here we have successfully utilized this approach to provide novel insights into α-syn function and toxicity, however; previously published research has yielded discrepant findings. For example, no toxicity or inflammation was reported in two independent studies in which endogenous α-syn was silenced in the mouse hippocampus or the squirrel monkey SNc (Lewis et al., 2008; McCormack et al., 2010). Other laboratories have reported an intermediate degree of toxicity (e.g., loss of striatal DA; Zharikov et al., 2015), likely reflecting functional deficiencies (Cali et al., 2012), following acute α-syn silencing. In contrast, we and others report robust nigral cell death following α-syn knockdown in the rat, mouse, and non-human primate using both shRNA and microRNA technology (Gorbatyuk et al., 2010; Khodr et al., 2011, 2014; Benskey et al., 2016a; Collier et al., 2016). Finally, the absence of endogenous α-syn during development leads to decreased numbers of DA neurons in the mouse SNc (Garcia-Reitboeck et al., 2013). We believe that the source of discrepancy between these findings is a differing degree of α-syn knockdown, the kinetics of α-syn knockdown, and the type of cell being targeted. Here we show a dose dependent effect of α-syn knockdown, where the degree of nigrostriatal toxicity is inversely proportional to the amount of endogenous α-syn remaining within the affected neurons (Gorbatyuk et al., 2010). Thus, it is likely that alternative reports, which did not report toxicity, did not reach the critical threshold of α-syn knockdown. For example, the degree of α-syn knockdown reported in studies that did not observe toxicity ranges between 35%–55% (Lewis et al., 2008; McCormack et al., 2010; Zharikov et al., 2015). In contrast, knockdown of α-syn by 70%–90% causes severe nigral cell loss (Gorbatyuk et al., 2010). These findings are in line with the level of knockdown and toxicity observed here. A ~50% reduction of α-syn was achieved following expression of the α-syn shRNA (FULL), whereas the α-syn shRNA (HALF) caused an initial ~25% reduction in α-syn, that eventually progressed to a ~50% reduction over the course of the study. Corresponding to the differing degrees of α-syn knockdown between the two titers used, the α-syn shRNA (FULL) caused a progressive loss of TH+ neurons beginning at 14 days post-injection, while expression of the α-syn shRNA (HALF) did not produce any detectable TH+ cell loss until 21 days post-injection. An important caveat to these observations is that these are the levels of knockdown observed within nigral neurons that still retain their TH phenotype. Due to technical reason (loss of GFP reporter expression at later time points) we were unable to quantify α-syn knockdown after loss of TH expression. As loss of TH expression is one of the first pathological changes we observed following α-syn knockdown, it is likely that 50% depletion is the critical threshold of α-syn knockdown, below which a pathological cascade is initiated. Taken together, it seems that in order for nigral pathology to occur, levels of endogenous α-syn must be decreased below a certain level (likely below 50%) and this depletion must be maintained for a certain period of time (likely at least 7 days). Finally, an important limitation to our study, and others, is the fact that we are using α-syn knockdown at the level of the soma as a surrogate for estimating α-syn levels at the synapse, the presumptive functional location of α-syn. However, it is extremely difficult to accurately measure nigral terminal α-syn in the striatum given the large number of striatal terminals originating from non-nigral areas.

### The Specificity of α-syn shRNA Mediated Toxicity

Nigrostriatal toxicity induced by shRNA-mediated α-syn knockdown is partially rescued by co-expression of a rat α-syn transgene rendered insensitive to the shRNA (Gorbatyuk et al., 2010). Although these data demonstrate that nigrostriatal cell loss is, at least in part, due to loss of endogenous α-syn, there is always a concern of non-specific toxicity when using RNAi. One goal of this work was to ensure that α-syn shRNA toxicity is indeed due to loss of α-syn function, and not an artifact from RNAi. To this end we examined the effects of α-syn silencing within WT and α-syn KO mice. If the toxicity we observe following expression of the α-syn shRNA is due to non-specific RNAi toxicity, than one would predict comparable toxicity within both WT and α-syn KO mice. If however, α-syn shRNA expression produces neurodegeneration by specifically decreasing endogenous α-syn, then α-syn KO mice should be immune to α-syn shRNA toxicity, as they do not express α-syn. α-syn KO mice were immune to the effects of α-syn shRNA administration, thus in conjunction with previously published controls (Gorbatyuk et al., 2010), we confidently conclude that α-syn knockdown-mediated nigral toxicity is solely mediated by loss of endogenous α-syn.

### Neuroinflammation Induced by α-syn Knockdown

In the work of Gorbatyuk et al. (2010), the integrity of the nigrostriatal system was first examined at 4-weeks post-shRNA administration, at which point there was an approximate 90% reduction of endogenous α-syn and an 80% loss of TH+ nigral neurons. Here we sought to determine if intra-neuronal α-syn dysfunction, in the absence of overt cell death, could elicit a neuroinflammatory response, thus we extensively characterized the early events in the pathological process that occur following loss of α-syn from nigrostriatal neurons, but prior to neurodegeneration.

One of the most remarkable changes we observed following α-syn knockdown was an up-regulation of neuronal MHC-1 expression, coinciding with the recruitment of immune cells. Members of the MHC-1 family are expressed on virtually all nucleated cells. However, under normal conditions, neuronal expression of MHC-1 is relatively low, especially in the adult brain (Boulanger and Shatz, 2004). Neuronal MHC-1 expression is suggested to play a role in synaptic plasticity (Corriveau et al., 1998), learning and memory (Nelson et al., 2013), and following injury (Boulanger and Shatz, 2004; Oliveira et al., 2004; Cebrián et al., 2014a). Additionally, neuronal MHC-1 expression is associated with neurodegenerative disorders such as Huntington’s disease, amyotrophic lateral sclerosis, multiple sclerosis and Parkinson’s disease (Boulanger and Shatz, 2004; Cebrián et al., 2014a, b). Within Parkinson’s disease, midbrain catecholaminergic neurons express MHC-1, while cultured midbrain DA neurons induce MHC-1 expression in response to increased cytosolic DA, oxidative stress, α-syn and cytokines released from glia. Finally, increased MHC-1 expression on cultured DA neurons triggers an antigenic response, leading to a cytotoxic T-cell-mediated death of DA neurons (Cebrián et al., 2014b).

Here we observed a near identical sequence of events following α-syn knockdown within DA neurons of the rat SNc, including neuronal MHC-1 expression, glial cell recruitment, and infiltration of T-cells. Although we observed an infiltration of T-cells into the brain of animals injected with the α-syn shRNA, it is currently unclear whether the T-cells were CD8+ cytotoxic T-cells or CD4+ helper T-cells. Previous work indicates that MHC-1 expression on catecholaminergic neurons results in the recruitment of CD8+ cytotoxic T-cells, which subsequently mediate the selective degeneration of the MHC-1+ neurons (Cebrián et al., 2014b). Although, this type of CD8+ T-cell response is in line with what we observe here, the identity of the infiltrating T-cells remains unknown. Nonetheless, the nature of the T-cell response elicited by α-syn knockdown in nigral neurons is very important, as is could provide a mechanism of cell death, and as such will be the topic of future investigation.

Examining the temporal sequence of increased neuronal MHC-1+ numbers vs. total MHC-1 expression suggests that there is a biphasic induction of MHC-1 expression within more than one cell type, specifically neurons and microglia. Neuronal MHC-1 expression increased at 10-days, peaked at 14 days, and decreased at 21-days post injection. The decrease in MHC-1+ neurons at the 21-day time point is likely due to the death of MHC-1 expressing nigral neurons. In contrast, total MHC-1 expression within the injected SNc progressively increased from 10 days to 21 days post-injection. This progressive increase in total MHC-1 expression, in the face of decreasing numbers of MHC-1+ neurons, is the result of a dramatic increase in the number of reactive microglia expressing MHC-1 within the injected midbrain.

From this sequence of events we believe that neuronal MHC-1 expression, and the recruitment of immune cells, is not merely a generalized inflammatory reaction to cell death, but rather a cell-autonomous response to loss of α-syn function, which actively contributes to neurodegeneration. For instance, MHC-1 was up-regulated within transduced neurons 10 days post-injection, a time point that precedes any detectable amount of cell death by almost 2 weeks. At these early time points, the majority of MHC-1 expressing neurons were located in the ventral tier of the injected SNc, the anatomical location exhibiting the greatest cell loss at 21 days post-injection, and the anatomical location that where the earliest signs of pathology are seen in Parkinson’s disease (Gibb and Lees, 1991). Finally, due to the fact that we silenced endogenous α-syn expression, and the inflammatory response occurred prior to cell loss, it is highly unlikely that the inflammation was initiated by a direct exposure of microglia to extracellular α-syn. We believe that these observations are consistent with microglial-mediated phagocytosis of stressed, yet still living, transduced neurons. Taken together, these data support an immune cell mediated process of neurodegeneration that is initiated by neurons, following intra-neuronal α-syn dysfunction.

Nonetheless, the initial impetus leading to MHC-1 up-regulation remains unclear. Neuronal expression of MHC-1 can be induced by viral infection (Redwine et al., 2001), however there was no increase in MHC-1 expression within AAV-myocardin shRNA transduced neurons, excluding this possibility. Further, previous work has demonstrated that a single administration of AAV is not sufficient to cause an inflammatory response (Peden et al., 2004). As mentioned, increased cytosolic DA (presumably leading to increased oxidative stress) can initiate neuronal MHC-1 up-regulation (Teoh and Davies, 2004; Cebrián et al., 2014b). α-syn regulates many aspects of chemical neurotransmission and DA biosynthesis (reviewed in Benskey et al., 2016a), thus, we predict that loss of normal α-syn function within nigral neurons results in a dysregulation of DA synthesis and handling, thereby increasing oxidative stress and MHC-1 expression (Teoh and Davies, 2004; Caudle et al., 2007; Cebrián et al., 2014b).

However, there was also a second initiation of MHC-1 expression at the 21-day time point, where non-transduced, TH+ neurons expressed MHC-1. This is likely caused by increased pro-inflammatory cytokine production within the injected SNc. Microglial-released pro-inflammatory cytokines, such as interferon gamma, are sufficient to induce MHC-1 expression in DA neurons (Cebrián et al., 2014b). Thus, it is likely that the large increase in reactive microglia observed at the 21 day time point, resulted in a corresponding increase in inflammatory cytokine production, thereby inducing MHC-1 expression within the remaining, un-transduced nigrostriatal neurons. Future research is needed to confirm this hypothesis, however, this mechanism could account for the continued nigral degeneration (80% loss of TH+ neurons) observed at the 1-month time point by Gorbatyuk et al. (2010).

### Nigrostriatal Pathology Induced by α-syn Knockdown

In parallel with a major immune response, α-syn knockdown also caused nigral neuron dysfunction, in the form of a progressive loss of TH expression. The loss of TH+ neurons was dose-dependent, indicating that the degree of TH neuron loss is proportional to the degree of α-syn knockdown. Remarkably, despite early losses in TH immunoreactivity, striatal DA concentrations were unaffected until the 21-day time point. This may be the result of disinhibition of TH following α-syn knockdown. α-syn acts as a negative regulator of both TH and aromatic amino acid decarboxylase (AADC; Perez et al., 2002; Perez and Hastings, 2004; Yu et al., 2004; Peng et al., 2005; Tehranian et al., 2006; Wang et al., 2009; Benskey et al., 2016a). Thus, α-syn knockdown likely disinhibits TH and AADC, resulting in increased *de novo* DA synthesis, maintaining normal DA levels in the face of decreased TH expression. Finally, 21-days following expression of the α-syn shRNA (Half), there was no change in the levels of striatal VMAT, despite an overall reduction in the number of TH+/HUc+ neurons in the ipsilateral SNc. This is likely the effect of collateral axonal sprouting of the nigrostriatal terminals remaining in the striatum, a phenomenon that occurs following striatal denervation which has been well documented in rodent models of Parkinson’s disease (Arkadir et al., 2014).

Interestingly, α-syn overexpression results in a near identical decrease in both TH expression (Baptista et al., 2003; Yu et al., 2004; Alerte et al., 2008; Li et al., 2011). Increased oxidative stress can cause the addition of carbonyl adducts to the TH protein, resulting in a loss of immunoreactivity (Dela Cruz et al., 1996). As such, the proposed (unregulated) increase in DA synthesis following α-syn manipulation, may result in DA auto-oxidation and a corresponding loss of TH protein following the addition of oxidative adducts. It is possible that both α-syn overexpression and silencing induce toxicity through a common mechanism, which is the loss of α-syn function. For example, α-syn overexpression, and subsequent aggregation, may produce toxicity by sequestering functional forms of endogenous α-syn into aggregates, causing a *de facto* loss of function (Perez and Hastings, 2004; Benskey et al., 2016a). From this viewpoint it is not surprising that both α-syn overexpression and knockdown result similar decreases in TH expression, albeit over a different time course. This theory is supported by α-syn based animal models where overexpression of α-syn, or injection of α-syn recombinant protein (preformed fibrils) increase α-syn aggregation, with a corresponding depletion of soluble α-syn (Perez et al., 2002; Perez and Hastings, 2004; Unni et al., 2010; Volpicelli-Daley et al., 2011; Cali et al., 2012; Osterberg et al., 2015). Interestingly, administration of α-syn PFFs results in an inflammatory response, similar to that observed here and in the PD brain, potentially suggesting that loss of normal α-syn function following PFF-induced aggregation can elicit an inflammatory response (Harms et al., 2017).

### Is Toxicity Induced by α-syn Dysfunction Exclusive to Dopaminergic Neurons?

One important question of the current study was whether intra-neuronal α-syn dysfunction within non-dopaminergic neurons could elicit neuroinflammation and toxicity. As mentioned above we propose that loss of α-syn function may mediate toxicity via impaired DA metabolism and handling. Indeed several reports indicate that toxicity initiated through manipulation of α-syn is dependent upon the presence of intracellular DA (Xu et al., 2002). Further, antibodies isolated from the sera of Parkinson’s disease patients react with proteins modified by DA oxidation. In line with these findings, no inflammation or neurodegeneration was detectable following α-syn knockdown within the cerebellum. Although this is by no means an exhaustive analysis of the effects of α-syn silencing in the myriad of non-dopaminergic neurons present in the brain, this result does support the notion that the inflammation and toxicity observed following α-syn dysfunction is specific to catecholaminergic neurons.

Interestingly, there was a phenotypic loss of calbindin expression within purkinje cells. This result was unanticipated due to the fact that there was no transduction of the PCL, and the fact that purkinje cells do not express appreciable amounts of α-syn (Lee et al., 2015). We believe that this loss of calbindin expression is the result of altered synaptic input from α-syn shRNA transduced granule cells onto purkinje cells. α-syn has been proposed to act as a negative regulator of synaptic transmission, and increasing synaptic input onto purkinje cells results in a decrease in calbindin mRNA and protein (Barmack and Qian, 2002). Thus, it is likely that removing α-syn from presynaptic granule cells increases neurotransmission at the granule-to-purkinje cell synapse, resulting in an activity dependent decrease in calbindin expression.

### Is Loss of α-syn Function an Early Event in Parkinson’s Disease Pathogenesis?

Based on the correlations between the data presented here and the sequence of pathological events observed in Parkinson’s disease, we believe that α-syn loss-of-function toxicity may, at least in part, contribute to neurodegeneration in Parkinson’s disease. Parkinson’s disease pathology is characterized by a phenotypic loss of TH preceding the death of nigral neurons (Kastner et al., 1993, 1994; Kordower et al., 2013), a significant inflammatory response involving microgliosis, neuronal MHC-1 expression, and infiltration of peripheral leukocytes to the brain (McGeer et al., 1988a, b; Croisier et al., 2005; Orr et al., 2005; Ouchi et al., 2005; Cebrián et al., 2014b). Within the above experiments we describe a pathological cascade that entails all of these pathological features following acute α-syn knockdown within nigral neurons. Further, there is ample evidence to suggest that loss of normal α-syn function contributes to Parkinson’s disease pathogenesis. For example, most familial SNCA mutations increase the aggregation kinetics of α-syn (Conway et al., 1998; Burré et al., 2010; Ghosh et al., 2014), while increasing cellular concentrations of α-syn (as is achieved with gene duplication) also increases the propensity of α-syn to aggregate (Conway et al., 1998; Uversky, 2007). As described above, the aggregation of α-syn could act to sequester functional forms of the protein, impairing normal function. This idea is supported by the fact that, although the brains of synucleinopathy patients do show accumulation of excess α-syn, the majority of α-syn is abberantly folded and/or contained within aggregates (Miller et al., 2004; Kramer and Schulz-Schaeffer, 2007; Quinn et al., 2012). Further, the brains of idiopathic Parkinson’s disease patients have decreased levels of soluble (presumably functional) α-syn (Baba et al., 1998; Quinn et al., 2012). In addition, most mutations in the SNCA gene directly impair the ability of the mutant protein to perform its normal function (Fortin et al., 2004; Burré et al., 2010; Fares et al., 2014; Logan et al., 2017; Pozo Devoto et al., 2017), again linking loss of α-syn function to neurotoxicity. Finally, Parkinson’s disease patients carrying the low repeat REP1 allele, which results in decreased SNCA expression (Chiba-Falek and Nussbaum, 2001), have worse motor and cognitive disease outcomes (Markopoulou et al., 2014). Viewed as a whole, we believe it is possible that an initial loss of function following α-syn aggregation in the Parkinsonian brain could elicit an early inflammatory response that contributes to neurodegeneration. Thus, examining the early pathological events following loss of α-syn function could provide insights into Parkinson’s disease pathogenesis as well as the identification of novel therapeutic targets. Further, these data provide further support for examining early inflammatory markers as potential biomarkers for PD. Indeed, a recent study was able to identify a panel of inflammatory markers isolated from human serum or cerebrospinal fluid that could reliably distinguish between healthy controls and PD patients (Eidson et al., 2017).

Finally, these data suggest that therapeutics aimed at decreasing or ablating α-syn within Parkinson’s disease-affected neurons should be undertaken with caution. From the current work it is clear that lowering α-syn levels beyond a critical threshold within nigral neurons can have deleterious consequences. Although it is theoretically possible to titer the degree of α-syn knockdown to a level that would be therapeutic in the context of PD, from a technical standpoint this could be extremely challenging if not impossible. We propose that a better therapeutic approach would be drugs or small molecules that prevent α-syn aggregation, or conversely, disassemble fibrilized α-syn to soluble, functional α-syn.

## Author Contributions

FPM conceived the experiments and performed surgery. MJB performed surgery, the experimentation, the data analysis and interpretation and wrote the manuscript. RCS performed stereological cell counting and surgery. IMS performed surgery. CES performed surgery and provided critical feedback on the manuscript. JWL performed HPLC analysis of neurochemicals.

## Conflict of Interest Statement

The authors declare that the research was conducted in the absence of any commercial or financial relationships that could be construed as a potential conflict of interest.
