# Supplementary material for: Silencing Alpha Synuclein in Mature Nigral Neurons Results in Rapid Neuroinflammation and Subsequent Toxicity
Source: Front Mol Neurosci. 2018 Feb 13;11:36. doi: 10.3389/fnmol.2018.00036 (PMC5819572; doi:10.3389/fnmol.2018.00036)
Supplement: Supplementary file 1 [file Image_1.pdf]

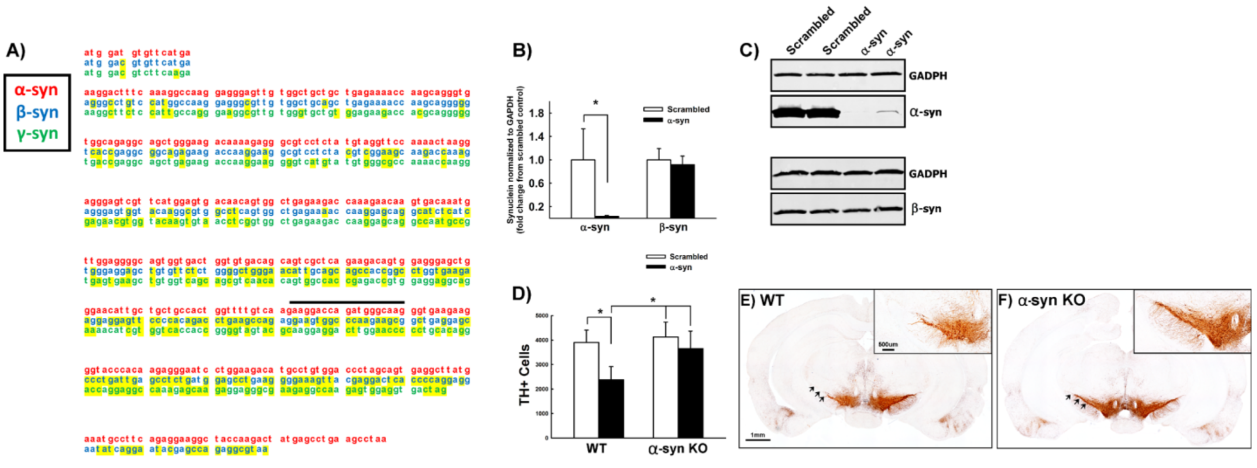

Supplemental Figure 1. α-syn knockout mice are immune to α-syn knockdown toxicity. (**A**) Alignment of the cDNA sequences of alpha-syn (α-syn; red), beta-synuclein (β-syn;blue) and gamm-synuclein (γ-syn; green). Sequences from β- and γ-syn that are not homologous with α-syn are highlighted in yellow. The position of the target α-syn siRNA is denoted as a black bar. (**B-C)** PC12 cells were transduced with lentivirus expressing the α-syn shRNA or a scrambled control shRNA. Quantitative western blotting was performed to measure levels of α-syn and β-syn within PC12 cells 5 days following transduction. Columns in panel **(B)** represent mean levels of α-syn or β-syn normalized to GAPDH, +1 SEM (n=5-8 replicates/group), expressed as fold-change from scrambled shRNA treated controls. Panel (**C**) shows representative blots of α-syn, β-syn and GAPDH. Membranes were cut to allow the use of multiple primary antibodies of the same host species. (**D-F**) Wild type (WT; C57bl/6j) or α-syn knockout (KO) mice received unilateral injections of AAV2/5 ($3x10^{13}$ vg/ml) expressing the target α-syn shRNA or a scrambled control shRNA into the SNc. One-month post-surgery mice were sacrificed, and numbers of tyrosine hydroxylase positive (TH+) neurons in the SNc were estimated using unbiased stereological cell counting. Columns in panel **(D)** represent mean number of TH+ cells in ipsilateral (injected) SNc, +1SEM (n=5-6/group), in animals receiving a scrambled control shRNA (**black columns**) or the α-syn shRNA (**white columns**). * Significantly different (p<0.05; t-test in panel B, ANOVA in panel D). Representative images of TH positive neurons of the SNc of WT and α-syn KO mice are shown in panels **(E-F)**. Insets in panels **(E-F)** represent high magnification images of the injected SNc delineated by black arrows. Scale bar in panel (**E**) represents 1mm and applies to panel (**F**). Scale bar in the inset in panel (**E**) represents 500μm and applies to the inset in panel (**F**).

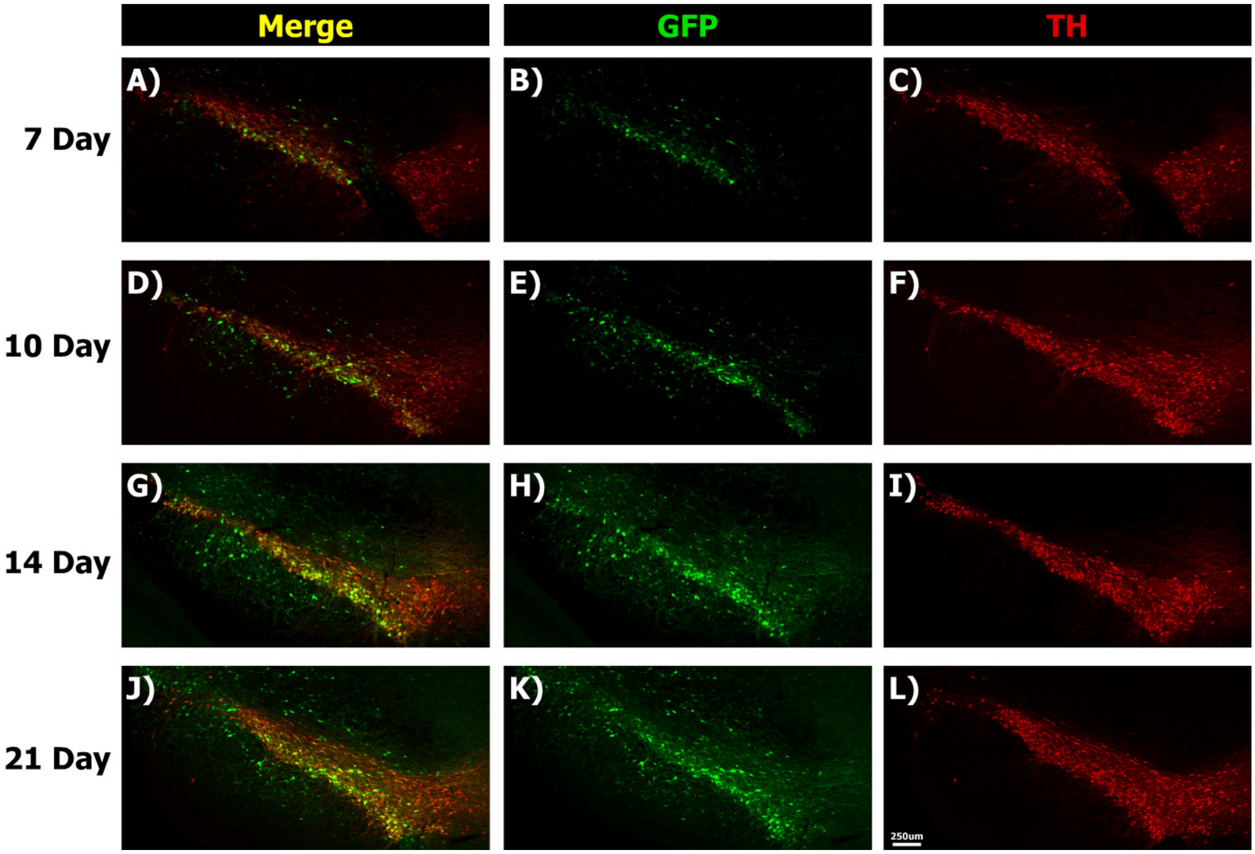

Supplemental Figure 2. Time course of AAV-mediated transduction within the SNc. Rats received a single injection of AAV2/5 (2.6×10^{12} vg/ml; Full) expressing a myocardin control shRNA and a GFP reporter transgene, and were sacrificed at 7, 10, 14, and 21 days post-surgery. Representative images depict expression of the GFP reporter gene (green) within TH+ neurons (red) at 7 (**A-C**), 10 days (**D-F**), 14 days (**G-I**) and 21 days (**J-L**) post-surgery. Scale bar in panel (**L**) represents 250µm and applies to panels (**A-K**).

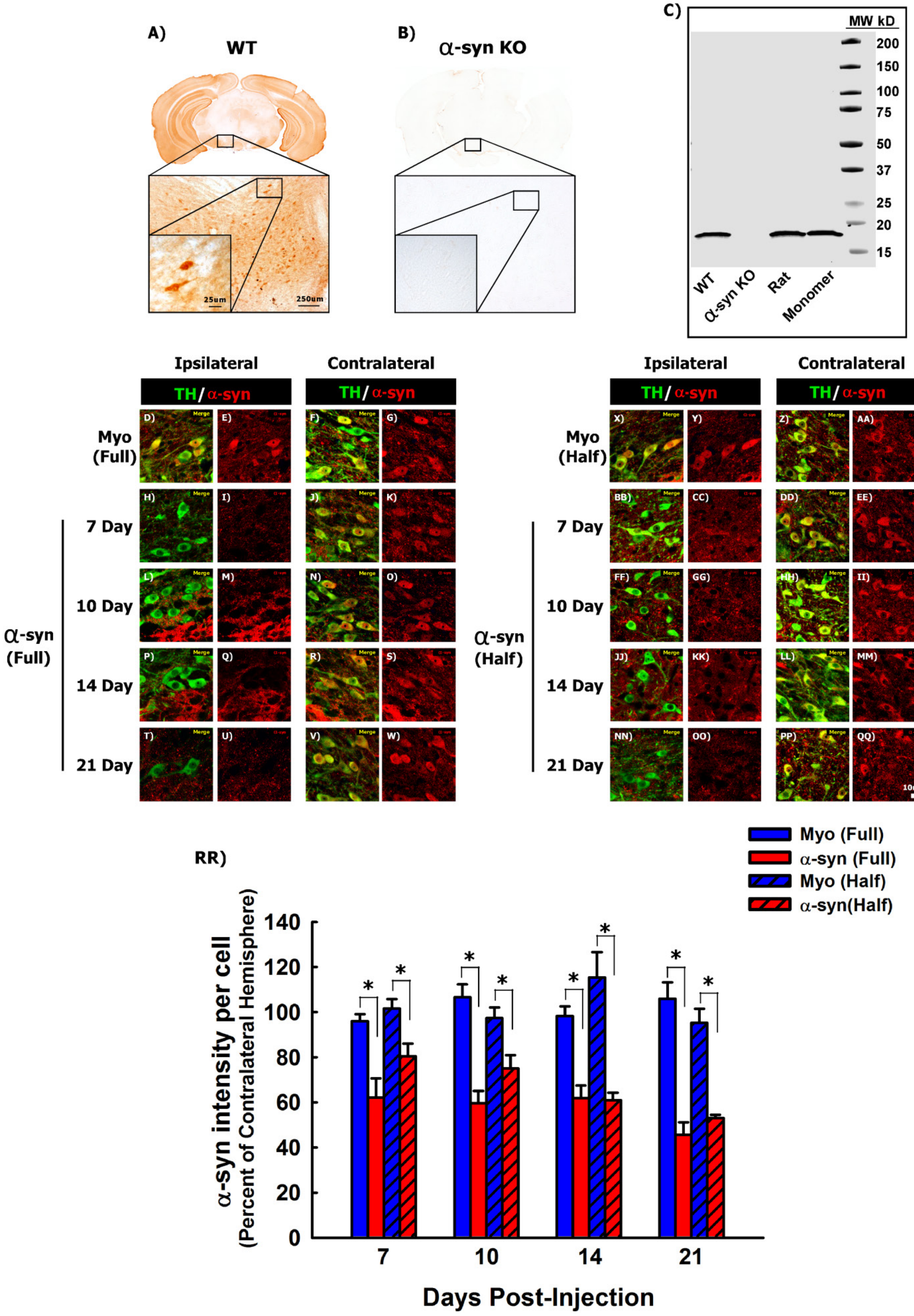

A)
WT
25um
250um
B)
α-syn KO
C)
MW kD
200
150
100
75
50
37
25
20
15
WT
α-syn KO
Rat
Monomer
Ipsilateral
Contralateral
TH/α-syn
Myo (Full)
7 Day
10 Day
14 Day
21 Day
α-syn (Full)
Myo (Half)
α-syn (Half)
10um
RR)
Myo (Full)
α-syn (Full)
Myo (Half)
α-syn(Half)
α-syn intensity per cell (Percent of Contralateral Hemisphere)
0
20
40
60
80
100
120
140
7
10
14
21
Days Post-Injection

Supplemental Figure 3. Quantification of α-syn knockdown. (A-B) shows representative images of brain tissue from wildtype (WT; C57bl6j) or α-syn knockout (KO) mice stained with the mouse-anti-α-syn antibody. (**C**) Western blotting with the mouse-anti-α-syn antibody of strital lysates of WT mice (lane 1), KO mice (lane 2), rat (lane 3), or recombinant monomeric α-syn protein (lane 4). (**D-QQ**)Rats received a single injection of AAV2/5 at a titer of $2.6x10^{12}$ vg/ml (Full) or $1.3x10^{12}$vg/ml (HALF) expressing a target α-syn shRNA or a myocardin control shRNA. Rats were then sacrificed at 7, 10, 14, and 21 days post-surgery, and high magnification confocal images of TH positive neurons (Green) in the ipsilateral and contralateral SNc were obtained, after which the fluorescence intensity of α-syn (red) was quantified using Nikon NES software. Columns in Panel **(RR)** represent mean fluorescent intensity per cell (calculated as mean cell intensity per hemisphere), expressed as percent of the contralateral hemisphere + 1 SEM (n=3-4/group) following expression of myocardin and α-syn shRNA Representative images of α-syn immunoreactivity within TH positive neurons is shown for myocardin or α-syn FULL (**D-W**) or myocardin and α-syn shRNA HALF (**X-QQ**). There was no change in α-syn immunoreactivity in any myocardin group, thus only the 21-day time points are shown. * Indicates significantly different (ANOVA: $P<0.001$). Scale bar in panel (**QQ**) represents 10μm and applies to panels (**D-PP**).

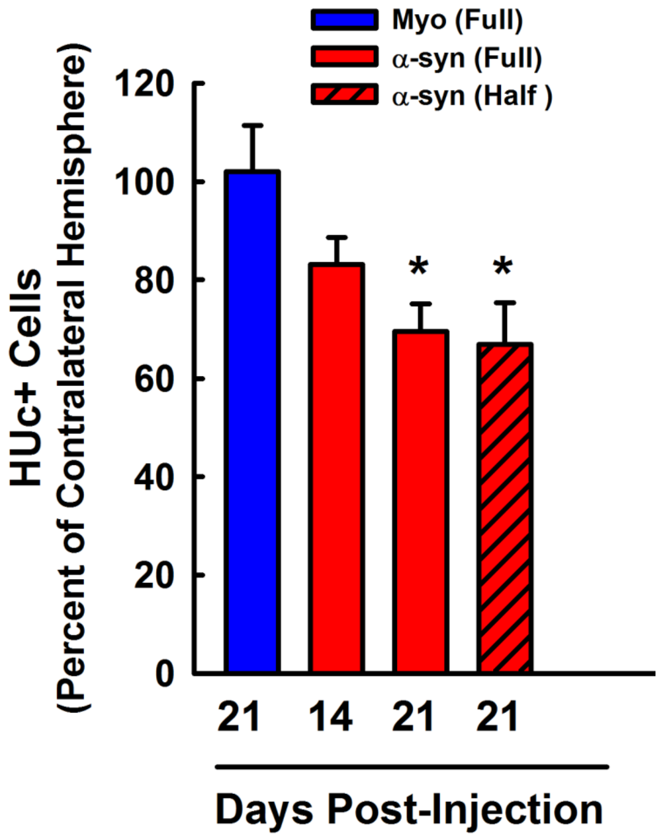

Supplemental Figure 4. The effect of α-syn knockdown on the total number of neurons in the SNc. Rats received unilateral injections of AAV2/5 expressing a target α-syn shRNA or a myocardin (Myo) control shRNA. AAV2/5 expressing the respective shRNAs was injected at a titer of 2.6×10^{12} vg/ml (Full) or 1.3×10^{12} vg/ml (Half). The number of cells expressing the pan-neuronal marker HUc (HUc+) in the SNc was estimated using unbiased stereological cell counting. Columns represent the mean number of HUc+ cells in the injected SNc, expressed as the percent of the contralateral hemisphere. HUc+ cells were only counted in experimental groups that showed a decrease in tyrosine hydroxylase positive cells in the injected SNc, i.e. 10, 14 and 21 days post α-syn shRNA (Full; **red bars**), or 21 days post α-syn shRNA (Half; **red hatched bar**). We detected no change in tyrosine hydroxylase positive or HUc+ cell numbers in the SNc at any time point following expression of the Myo shRNA, thus HUc+ cells from the Myo shRNA treated animals are presented as a single time point (Myo Full; **blue bar**). * Indicates significantly different than the Myo shRNA group (ANOVA: $p<0.05$).

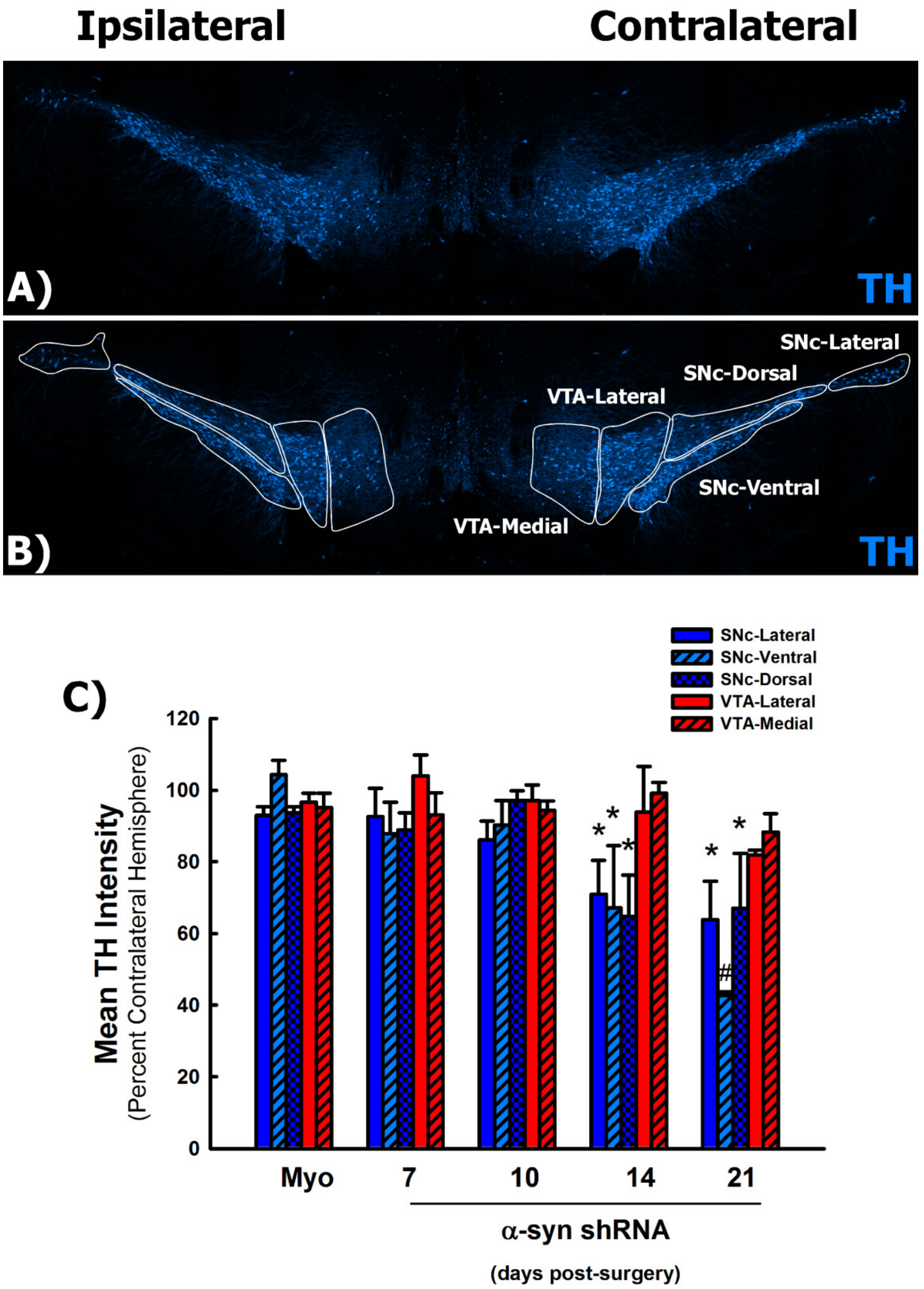
Ipsilateral
Contralateral
A)
TH
B)
SNc-Lateral
SNc-Dorsal
VTA-Lateral
SNc-Ventral
VTA-Medial
TH
C)
SNc-Lateral
SNc-Ventral
SNc-Dorsal
VTA-Lateral
VTA-Medial
Mean TH Intensity
(Percent Contralateral Hemisphere)
120
100
80
60
40
20
0
Myo
7
10
14
21
α-syn shRNA
(days post-surgery)

Supplemental Figure 5. α-syn silencing within nigrostriatal neurons decreases TH expression. Rats received a single injection of AAV2/5 (2.6x10^{12} vg/ml; Full) expressing a target α-syn shRNA or a myocardin (Myo) control shRNA. Rats were then sacrificed at 7, 10, 14, and 21 days post-surgery, and brains were removed, sectioned, and processed for immunohistochemical detection of tyrosine hydroxylase (TH). High magnification confocal images of TH positive neurons in the ipsilateral and contralateral SNc were obtained, after which the TH fluorescence intensity was quantified using Nikon NES software. In an effort to detect any sub-regional differences in TH intensity, midbrain dopamine neurons were subdivided into the medial ventral tegmental area (VTA-Medial), the lateral VTA (VTA-Lateral), the dorsal tier of the SNc (SNc-Dorsal), the ventral tier of the SNc (SNc-Ventral), and the lateral SNc (**A-B**). Columns in Panel **(C)** represent mean TH fluorescent intensity per cell (calculated as mean cell intensity per hemisphere), expressed as percent of the contralateral hemisphere, + 1 SEM (n=4-5/group). * Indicates significantly different (ANOVA: $p<0.05$) than all subdivisions of the Myo shRNA treated SNc and VTA. # Indicates significantly different (ANOVA:$p<0.05$) than all other groups.

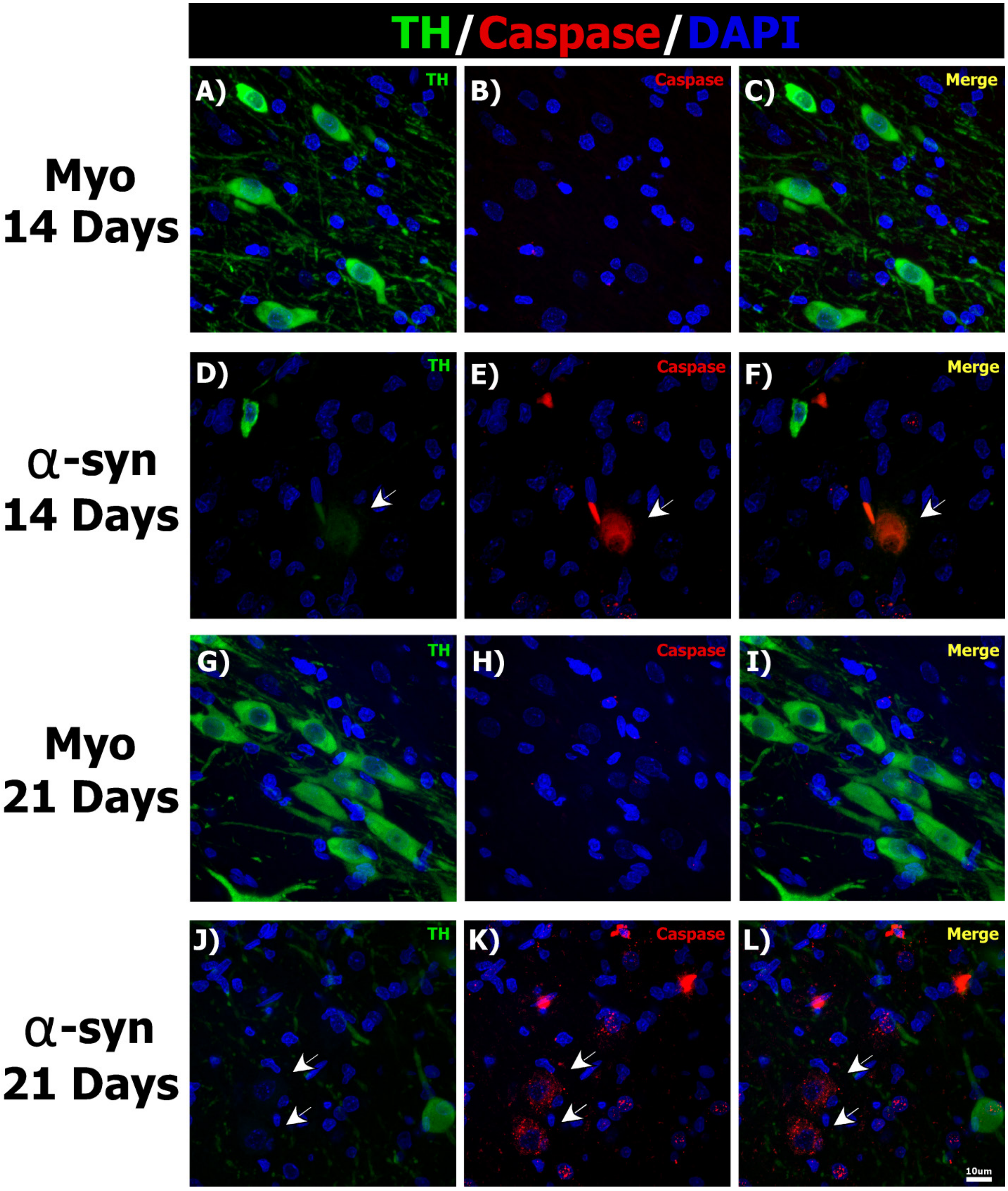
TH/Caspase/DAPI
Myo
14 Days
α-syn
14 Days
Myo
21 Days
α-syn
21 Days
A)
TH
B)
Caspase
C)
Merge
D)
TH
E)
Caspase
F)
Merge
G)
TH
H)
Caspase
I)
Merge
J)
TH
K)
Caspase
L)
Merge
10um

Supplemental Figure 6. α-syn silencing in nigrostriatal neurons increases levels cleaved caspase-3. Rats received a single injection of AAV2/5 (2.6x10^{12} vg/ml; Full) expressing a target α-syn shRNA or a myocardin (Myo) control shRNA. Rats were then sacrificed at 7, 10, 14, and 21 days post-surgery, and brains were processed for detection of tyrosine hydroxylase (TH; green) and cleaved caspase 3 (red). Extremely low levels of cleaved caspase-3 were detected in the SNc of myocardin shRNA treated animals at 14 days (**A-C**) or 21 days (**G-I**) or at the 7 or 10-day time point following α-syn shRNA expression (not shown). α-syn shRNA expression increased levels of cleaved caspase-3 at 14 **(D-F**) and 21 days (**J-L**) following injection. Most cleaved caspase-3 was observed in cells with neuronal morphology that were TH negative. Scale bar in panel **(L)** represents 10μm and applies to panels (**A-K**).

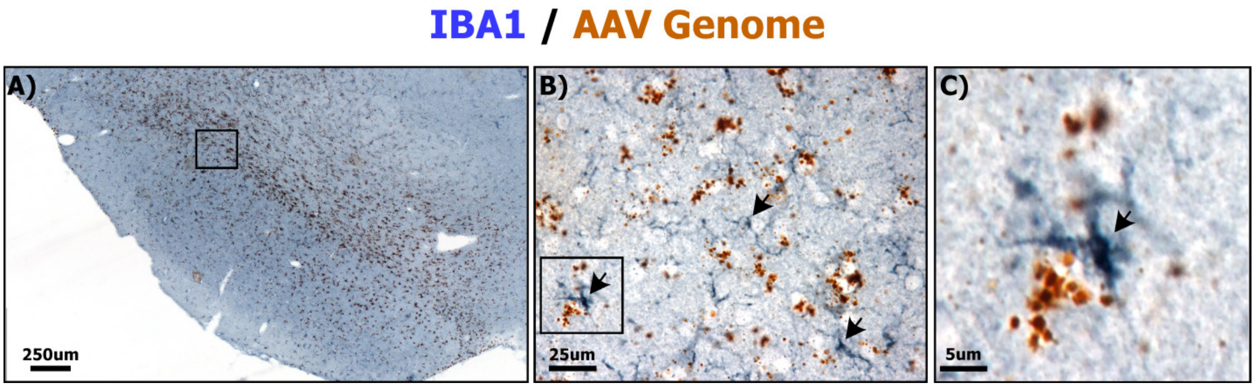

Supplemental Figure 7. AAV genomes are absent from microglia within the transduced SNc. Rats received a single injection of AAV2/5 (2.6×10^{12} vg/ml; Full) expressing the myocardin (Myo) control shRNA. Rats were then sacrificed 21 days post-surgery, and brains were removed, sectioned and processed for RNAscope *in situ* hybridization of the promoter within the AAV genome and immunohistochemical detection of IBA1 to visualize microglia. Representative images of *in situ* hybridization of the AAV genome (brown) and IBA1+ microglia (blue) are shown. **Arrows** indicate IBA1+ microglia that do not co-localize with the AAV genome. Panel (**B**) corresponds to the area within the box in panel (**A**). Panel (**C**) corresponds to the area within the box in panel (**B**). Scale bars represent 250µm, 25µm and 5µm in panels (**A**), (**B**) and (**C**) respectively.
